# Plant ingredients in Thai food: a well-rounded diet for natural bioactive associated with medicinal properties

**DOI:** 10.7717/peerj.14568

**Published:** 2023-03-01

**Authors:** Raveevatoo Buathong, Sutsawat Duangsrisai

**Affiliations:** Department of Botany, Faculty of Science, Kasetsart University, Bangkok, Thailand

**Keywords:** Bioactive compounds, Thai plants, Food, Anti-inflammatory, Antibacterial, Antiviral agents

## Abstract

**Background:**

Seeking cures for chronic inflammation-associated diseases and infectious diseases caused by critical human pathogens is challenging and time-consuming. Even as the research community searches for novel bioactive agents, consuming a healthy diet with functional ability might be an effective way to delay and prevent the progression of severe health conditions. Many plant ingredients in Thai food are considered medicinal, and these vegetables, herbs, and spices collectively possess multiple biological and pharmacological activities, such as anti-inflammatory, antimicrobial, antidiabetic, antipyretic, anticancer, hepatoprotective, and cardioprotective effects.

**Methodology:**

In this review, the selected edible plants are unspecific to Thai food, but our unique blend of recipes and preparation techniques make traditional Thai food healthy and functional. We searched three electronic databases: PUBMED, Science Direct, and Google Scholar, using the specific keywords “Plant name” followed by “Anti-inflammatory” or “Antibacterial” or “Antiviral” and focusing on articles published between 2017 and 2021.

**Results:**

Our selection of 69 edible and medicinal plant species (33 families) is the most comprehensive compilation of Thai food sources demonstrating biological activities to date. Focusing on articles published between 2017 and 2021, we identified a total of 245 scientific articles that have reported main compounds, traditional uses, and pharmacological and biological activities from plant parts of the selected species.

**Conclusions:**

Evidence indicates that the selected plants contain bioactive compounds responsible for anti-inflammatory, antibacterial, and antiviral properties, suggesting these plants as potential sources for bioactive agents and suitable for consumption for health benefits.

## Introduction

Since the pandemic of Severe Acute Respiratory Syndrome Coronavirus-2 (SARS-CoV-2) began in 2019, searching for new potential antiviral agents and prophylactics has driven priorities in public health research and the scientific community. Furthermore, treatment failure, drug side effects, and resistance have broadly inspired a shift in direction to investigating natural plant products as sources of effective bioactive agents. This updates a previous compilation of 207 plant species reported as having antiviral activity against essential and clinically significant viruses such as human immunodeficiency virus (HIV), herpes simplex virus (HSV), influenza, and hepatitis C (Mohan et al., 2020b).

Edible plants have the advantages of featuring milder toxicity profiles and being easy to access. In Indian cuisine, 38 edible plant species used as food or nutraceuticals have shown anti-retroviral activity, some of which also possess anti-inflammatory, immunomodulatory, or angiotensin-converting-enzyme (ACE) inhibitory activity (Patel et al., 2021). Herbal medicine is also a potent source of compounds with therapeutic properties. Antioxidant and antiviral potential has also been demonstrated in a summary of 18 herbal antimicrobial agents including clove, portulaca, tribulus, eryngium, cinnamon, turmeric, ginger, thyme, pennyroyal, mint, fennel, chamomile, burdock, eucalyptus, primrose, lemon balm, mallow, and garlic (Parham et al., 2020). Some similar articles have also been published on selected plant species. For instance, *Allium cepa* (Amaryllidaceae) juice, methanolic extract, and constituents like allicin, quercetin, and allyl methyl disulfide have shown anti-inflammatory effects in different models and with different mechanisms of action (Marefati et al., 2021). A review article of different plant parts, extraction solvents, and seed oil of *Coriandrum sativum* (Apiaceae) experimentally demonstrated anti-inflammatory effects in macrophages, mice, and rats (Sari, Bellatasie & Ifora, 2021). A trypsin inhibitor protein from seed of *Tamarindus indica* (Fabaceae) is proposed to have utility for inhibiting proteases related to SAR-CoV-2 infection in the worsened inflammatory condition in obesity (de Morais et al., 2021). Curcumin from *Curcuma longa* (Zingiberaceae) is also proposed as a potential prophylactic therapeutic for COVID-19 due to its observed antiviral activity against ten types of enveloped viruses that cause human diseases combined with an immunomodulatory effect (Thimmulappa et al., 2021). Additionally, xanthones in many plant species, including *Garcinia cowa* (Clusiaceae), can potentially treat inflammatory skin diseases through different mechanisms (Gunter et al., 2020).

Alongside an active lifestyle, a healthy and functional diet is of interest for maintaining and promoting well-being to prevent, delay, and even treat ailments. That is, functional food may help to boost the immune system, reduce risk of noncommunicable diseases, and improve memory or physical condition (Topolska, Florkiewicz & Filipiak-Florkiewicz, 2021). Several edible and medicinal plants serve as daily elements of the diet in traditional Thai cuisine, which emphasizes aromatic components, flavor, and appearance and utilizes a range of preparation methods such as spicy salads, steaming or boiling, stir-frying, frying, and chili pastes for a dip. Moreover, Thai cuisine includes many coconut-milk-based curry pastes with slightly different compositions like red curry, sweet green curry, yellow curry, nutty curry, a fusion of Thai and Indian styles (Massaman curry), and non-coconut-milk based curry pastes such as the famous Tom Yam (hot and sour soup with or without coconut milk), Tom Kah (coconut milk with galangal rhizome and kaffir lime leaves), and Gaeng Som (sour fish curry paste soup) (Kanchanakunjara, Chantachon & Koseyayotin, 2017; Khanthapok & Sukrong, 2019). The unique combination of vegetables, herbs, and spices used in Thai dishes makes Thai food an excellent example of a healthy diet. Notably, many of these plants have also been used in traditional medicine over generations to maintain health or relieve and cure ailments and infectious diseases. With the increasing problems posed by antimicrobial resistance, viral diseases, and inflammatory conditions related to various symptoms and diseases, the ingredients and medicinal plants in Thai dishes could comprise a valuable potential resource for the discovery of bioactive compounds with anti-infective and protective effects.

Here, we highlight different plant species and parts used in Thai food and traditional medicine simultaneously, as part of a functional diet and as phytomedicine; we also discuss their important metabolites, therapeutic uses, and biological and pharmacological activities. Furthermore, this review summarizes an update of recent research papers on plant extracts and, in some instances, isolated compounds that have demonstrated anti-inflammatory effects and antibacterial activity against selected critical antibiotic-resistant bacteria (*Staphylococcus aureus*, *Escherichia coli*, *Pseudomonas aeruginosa*, *Klebsiella pneumoniae*) or antiviral activity against clinically significant human viruses such as influenza virus, herpes simplex virus, Zika virus, and SARS-CoV-2.

The present review provides an easy-to-access list of edible and medicinal plants as a database, which is vital in developing further applications in clinical practice and drug discovery to comply with the UN goal of sustainable development to promote good health and well-being. Moreover, these plants might be integrated into food processing and manufacturing or added into any diet regimen as ingredients that promote and restore health. Our review can guide further research and offers highly interesting material for other scientists working on several aspects of plant-related life sciences, natural products, and the international plant science community. Thai food is complex, and its plant ingredients are abundant in tropical and subtropical regions; that is, the plants discussed here are not specific to the South-East Asia region. To the best of our knowledge, our work represents one of the most comprehensive indexes of diverse plants from a single cuisine that feature bioactivities that impact human health. It can inform and increase public awareness about the importance of ongoing research activities in plant science, phytochemistry, and pharmacognosy.

## Survey methodology

For the development of this review, globally-recognized names of the plant materials selected based on their use in Thai food and folk medicine were obtained from Plants of the World Online (POWO; https://powo.science.kew.org/) and the International Plant Names Index (IPNI; https://www.ipni.org/). Related literature dated from 2017 to 2021 was then sourced from scientific databases including PUBMED, Science Direct, and Google Scholar by searching with the term “Plant name” followed by the keyword “Anti-inflammatory” or “Antibacterial” or “Antiviral.” After carefully considering the obtained literature, we took only those publications that fit our scope of review. We proceeded to develop our database and produce this review. The compiled plant species and associated phytochemicals presented here have demonstrated the most promising health benefits and advantages with regard to targeting lead compounds.

## An overview of culinary herbs, spices, and medicinal plants in thai food

We summarized a selection of 69 species (33 families) from edible and medicinal plants used in Thai food, and also *Zingiber cassumunar* in the family Zingiberaceae. The selection covers plant parts consumed as vegetables or as ingredients in the preparation of many Thai dishes. Based on several phytochemical studies, all of the selected plant species are potential sources of bioactive compounds in various classes; Table 1 presents the main chemical compositions of the different plant families and genera.

**Table 1:** Plants ingredient in Thai food.

Family	Botanical name	Local name	Food preparation	Part	Phytochemicals	References
Amaranthaceae	*Suaeda maritima* (L.) Dumort.	Cha kram ชะคราม	Fresh, boiled, seasoning	Leaves	Essential oil, fatty acids, campesterol (steroid), ethanone (phenolic compound)	Nayak et al. (2018)
Amaryllidaceae	*Allium ascalonicum* L.	Hom daeng หอมแดง	Flavoring or seasoning and one of the main components in all Thai curries	Bulb	Organosulfur compounds (allin, allicin), flavonoids, phenolic compounds (quercetin, kamferal)	Ounjaijean et al. (2018)
	*Allium cepa* L.	Hom yai หอมใหญ่	Fresh, flavoring	Bulb	Organosulfur compounds, polyphenols, saponins, flavonoids quercetin, quercetin glycosides, flavonols	Kothari, Lee & Kim (2020), Marefati et al. (2021)
	*A. cepa var. aggregatum G. Don*	Ton hom ต้นหอม	Fresh, flavoring, decorating	Bulblets, leaves	Volatile compound: allyl-propyl disulphide	Saraswathi et al. (2017)
	*Allium sativum* L.	Kra thiam กระเทียม	Flavoring agent in all types of Thai food and usually do not remove the peel entirely to remain the fragrance or used raw as thin slices	Bulb	Organosulfur compounds (alliin, allicin), γ-glutamyl cysteine derivatives	Shao et al. (2020), Rouf et al. (2020)
Apiaceae	*Apium graveolens* L.	Khuen chai คื่นช่าย	Flavoring	Leaves, stem	Phthalides and derivatives, flavonoids: quercetin, apigenin, chrysoeriol, luteolin; glycosides, steroids, alkaloids, furocoumarins, phenols, sesquiterpenes alcohol, phenolic acids, tocopherol, terpenoids, essential oils	Salehi et al. (2019b), Emad et al. (2020), Khairullah et al. (2021)
	*Centella asiatica* (L.) Urb.	Bua bok บัวบก	Fresh, beverage	Leaves	Triterpene asiaticoside, asiatic acid, madecassoside, madecassic acid	Sun et al. (2020), Hafiz et al. (2020)
Apiaceae	*Coriandrum sativum* L.	Phak chi ผักชี	Leaves and stems are eaten fresh, root is typically mixed with garlic and pepper, seed is used in making curry paste	Whole plant	Flavonoids, essential oil, tannins, phenolics, alkaloids, terpenoids, fatty acids, sterols, glycosides	Kothalawala et al. (2020), Sari, Bellatasie & Ifora (2021)
	*Cuminum cyminum* L.	Yira ยี่หร่า	Roasted seed to heighten fragrance before used as a flavoring and condiment in many curry dishes	Seed	Cuminaldehyde and cuminic alcohol, roasted seeds contain substituted pyrazines, 2-ethoxy-3-isopropylpyrazine, 2-methoxy-3-sec-butylpyrazine, 2-methoxy-3-methylpyrazine flavoalkaloids (flavonoid alkaloids)	Srinivasan (2018), Kang et al. (2019)
	*Eryngium foetidum* L.	Phak chi farung ผักชีฝรั่ง	Fresh, flavoring	Leaves	(E)-2-dodecenal, 13-tetradecenal, dodecanal, 2,4,5-trimethylbenzaldehyde	Thomas et al. (2017)
Apocynaceae	*Telosma* cordata (Burm. f.) Merr.	Khachon ขจร	Boiled, stir fired, sour curry paste soup	Flower buds	Phenolics, volatile components: geraniol, beta-ionone, dihydro-beta-ionone, dihydro-beta-ionol, cis-and trans-theaspirane	Nguyen (2020)
Basellaceae	*Basella alba* L.	Phak plung ผักปลัง	Boiled, soup	Leaves	Betacyanin, carotenoids, bioflavonoids, β-sitosterol, lupeol	Chaurasiya et al. (2021)
Caricaceae	*Carica papaya* L.	Malako มะละกอ	Spicy salad “Som Tum”, sour curry paste soup	Unripe fruit	Octadecanoic acid, hexadecenoic acid, hexadecanoic acid, methyl ester, enzymes: papain, chymopapain	Sharma et al. (2020)
Cleomaceae	*Cleome gynandra* L.	Phak Sian ผักเสี้ยน	Pickled vegetable	Leaves	Methyl glucosinolate (glucocapparine), flavonoids, triterpenoids, sitosterol	Adhikari & Paul (2018)
Clusiaceae	*Garcinia cowa* Roxb. ex Choisy	Cha moung ชะมวง	Leaves used in soup or stew	–	Prenylated and oxygenated xanthones	Santo et al. (2020)
	*Garcinia schomburgkiana* Pierre	Ma dan มะดัน	Fruits used in soup, chili paste	–	Bixanthones (in twigs), xanthones, biphenyls, flavonoids, benzoylphloroglucinols	Do et al. (2020)
Cucurbitaceae	*Coccinia grandis* (L.) Voigt	Tum lueng ตำลึง	Boiled, soup	Arial part, leaves	Alkaloids, saponins, flavonoids, phenols, tannins, heptacosane, cephalandrol, β-sitosterol, cephalandrins A and B	Harshitha, Prasanthi & Ramarao (2018)
	*Cucurbita moschata* Duchesne	Fak Thong ฟักทอง	Boiled, stir-fried, dessert	Pulp,	Gallic acid, protocatechuic acid, 4-hydroxybenzoic acid, vanillic acid, chlorogenic acid, caffeic acid, rutin, carotenoids, phenolic acids, flavonols	Kulczynski & Gramza-Michałowska (2019)
	*Momordica charantia* L.	Mara มะระ	Fresh vegetable, boiled, stir-fried	Fruit	Charantia (cucurbitane triterpenoids), α-momorcharin, MAP30, saponins, flavonoids, alkaloids, sterols	Villarreal-La Torre et al. (2020)
	*Lagenaria siceraria* (Molina) Standl.	Nam Tao น้ำเต้า	Boiled	Fruit	Cucurbitacin B, mucilage, sterols, terpenoids, flavonoids, saponins	Tyagi ,Sharma & Shrivastava (2017)
	*Luffa acutangular* (L.) Roxb.	Bob ream บวบเหลี่ยม	Boiled, stir-fried, soup	Unripe fruit	Flavonoids, anthraquinone, triterpenes, volatile components	Shendge & Belemkar (2018)
Dilleniaceae	*Dillenia indica* L.	Ma tad มะตาด	Flavoring	Fruit	Betulin (pentacyclic triterpenoid), betulinic acid, cycloartenone, sterols, glycosides, saponins	Barua, Yasmin & Buragohain (2018)
Euphorbiaceae	*Phyllanthus emblica* L.	Ma kham pom มะขามป้อม	Raw or mixed with chili paste	Fruit	Flavonoids, tannins, diterpenes, gallic acid, ellagic acid, corilagin, chebulagic acid, quercetin	Jantan et al. (2019)
Fabaceae	*Acacia pennata* (L.) Willd.	Cha om ชะอม	Fried, sour curry paste	Leaves	Flavonoids: apigenin, quercetin- and kaempferol diglycoside, isorhamnetin mono-glycoside, isovitexin, flavanol glycosides, terpenoids	Aye et al. (2019), El-Taher et al. (2021)
	*Clitoria ternatea* L.	Anchan อัญชัน	Colorant, beverage, fresh, stir-fried	Flower	Flavonol glycosides anthocyanins, rutin (flavone), epicatechin (flavanol); polyphenolic acids: gallic acid, protocatechuic acid, chlorogenic acid; ternatins A1-3, B1-4, C1-5, D1-3	Gollen, Mehla & Gupta (2018), Oguis et al. (2019)
	*Neptunia prostrata* (Lam.) Baill.	Phak kra ched ผักกระเฉด	Raw, boiled, stir-fried	Leaves	Pheophorbide a, phenolic compounds, derivatives of quercetin, kaempferol, apigenin	Sagolshemcha & Singh (2017), Lee et al. (2019)
	*Leucaena leucocephala* (Lam.) de Wit	Kra tin กระถิน	Raw, boiled	Leaves	Flavones, flavonols, flavanone, flavanonol, flavonol glycosides, 1,2-benzenedicarboxylic acid, mono (2-ethylhexyl) ester, betulin, lupeol, androstan-17-one,3-ethyl-3-hydroxy-, (5à)-, 9,12,15-octadecatrienoic acid, methyl ester, (Z,Z,Z)-, betamethasone, β-sitosterol	Xu et al. (2018), Zayed, Wu & Sallam (2019)
	*Parkia speciosa* Hassk.	Sataw สะตอ	Raw, stir-fried	Seeds	Cyclic polysulfides, 1,2,4-trithiolane, propanoic acid), 3,3-thiobis-didodecyl ester, phenols, flavonoids, alkaloids, terpenoids, fatty acids	Chhikara et al. (2018), Saleh et al. (2021)
	*Sesbania grandiflora* (L.) Pers.	Dok Kae ดอกแค	Boiled and sour curry paste	Flower	Flavonoids, tannins, anthraquinones, steroids, terpenoids	Mohiuddin (2019)
	*Senna siamea* (Lam.) H.S.Irwin & Barneby	Khilek ขี้เหล็ก	Boiled and then cooked with curry and coconut milk	Leaves	Polyphenols, anthraquinone, anthocyanins, alkaloids, cardiotonic glycosides, saponins, steroids, terpenoids	Ntandou et al. (2018)
Fabaceae	*Tamarindus indica* L.	Ma kham มะขาม	Unripe fruit juice used as flavoring in Pad Thai, sour curry paste soup, tamarind sauce	Fruit	Alkaloids, flavonoids, tannins, phenols, saponins, triterpenoids, fatty acids, steroids	Komakech et al. (2019)
Gnetaceae	*Gnetum gnemon* L.	Phak lueng ผักเหลียง	Stir-fried	Leaves	2,3-dihydroxypropyl icosanoate, oleic acid, ursolic acid, phenylheptanoid gnetumal, callyspinol, cassipourol, (+)-dehydrovomifoliol, *p*-coumaric acid, ferulic acid	Dutta et al. (2018), Le et al. (2020)
Hypericaceae	*Cratoxylum formosum* (Jacq.) Benth. & Hook.f. ex Dyer	Phak tiu ผักติ้ว	Soup	Leaves root	Vismiaquinone, naringenin and 2,3-trans-dihydro-kaempferol Xanthones, anthraquinones, triterpenes	Juanda et al. (2019), Rodanant et al. (2017)
Lamiaceae	*Mentha × cordifolia* Opiz ex Fresen.	Saranae สะระแหน่	Flavoring, raw, garnish	Leaves	Volatile oil: monoterpenoids like carvone, limonene, menthone, menthol, pulegone, dihydrocarveol, s-carvone	Sevindik (2018)
	*Ocimum sanctum* L.	Kaphrao กระเพรา	Flavoring	Leaves	Flavones (cirsilineol, circimaritin, isothymusin, apigenin), terpenoid (carvacrol and caryophyllene)	Kaur et al. (2020)
	*Ocimum gratissimum* L.	Yira ยี่หร่า	Flavoring, raw	Leaves	Essential oils, phenolic compounds: rutin, quercetin, caffeic acid, rosmarinic acid, circhoric acid, sitosterol, ursolic acid, salvigenin, transferulic acid; tannins, saponins, flavonoids, anthraquinone glycosides	Ajayi et al. (2017), Alabi et al. (2018)
	*Ocimum × africanum* Lour.	Maeng lak แมงลัก	Flavoring, raw	Leaves	Essential oils: linalool, eugenol, 1,8-cineole, and camphor	Marrelli et al. (2020)
	*Ocimum basilicum* L.	Horapha โหระพา	Flavoring, raw	Leaves	Linalool, methyl chavicol, eugenol, bergamotene, methyl cinnamate, flavonoids, steroids, saponins, tannin	Shahrajabian, Sun & Cheng (2020)
Lauraceae	*Cinnamomum verum* J.Presl	Ob chei อบเชย	Yellow curry, stew	Stem bark	Trans-cinnamaldehyde, *p*-cymene, eugenol, *o*-methoxycinna-maldehyde, cinnamyl alcohol, benzyl benzoate, cinnamic acid, coumarin, phenolics compounds	Schink et al. (2018), Parham et al. (2020)
Meliaceae	*Azadirachta indica* A. Juss. var. siamensis Valeton	Sadao สะเดา	Vegetable, boiled	Leaves,	Triterpenoids, nimbin, glycosides nimbanene, 6-desacetylnimbinene, nim-bandiol, nimbolide, ascorbic acid, n-hexacosanol, amino acid, 7-desacetyl-7-benzoylazadiradione, 7-desacetyl-7-benzoylgedunin, 17-hydroxyazadiradione, nimbioland; flavonoids, saponins, tannins, alkaloids, limonoids, catechins, sterols, gallic acid	Islas et al. (2020)
Menispermaceae	*Cissampelos pareira* L.	Khruea ma noi เครือหมาน้อย	leaf juice is rich of pectin used in cooking or jelly dessert	Leaves	Alkaloids: isoquinoline alkaloids, benzylisoquinoline alkaloids (lau-danosine), aporphines (nuciferine, corytuberine, bulbocarpine, *nor-N-*magnoflorine, flavonoids, fatty acids	Iram et al. (2017), Kumari et al. (2021)
	*Tiliacora triandra* Diels	Yanang ย่านาง	Boiled with bamboo shoots and eaten as vegetable	Leaves	Phenolic compounds: gallic acid, cyanidin, quercetin, catechin; hydrocarbon compounds: phytol, 1-cyclohexenylacetic acid, oleamide, oleic acid	Weerawatanakorn et al. (2018), Pasachan et al. (2021)
Moringaceae	*Moringa oleifera* Lam.	Ma rum มะรุม	Leaves and pods can be boiled, stir-fried, and soup	Leaves seeds	Kaempferol, gallic acid, vanillic acid, coumaric acid, quercetin Isothiocyanate-1, glycosidic glucosinolates, isothiocyanates, nitriles, carbamates, thiocarbamates	Saleem, Saleem & Akhtar (2020) Jaja-Chimedza et al. (2017)
Musaceae	*Musa × paradisiaca* L.	Hua Plee หัวปลี	Banana blossom is consumed raw as vegetable with “Pad Thai” and cooked	Flowers	Phenolics, flavonoids, saponins	Shubham et al. (2019)
Myristicaceae	*Myristica fragrans* Houtt.	Luke Chan thet ลูกจันทน์เทศ	Flavoring, Thai Mussaman curry	Seeds	Essential oils, terpene hydrocarbons (sabinene, pinene, camphene, p-cymene, phellandrene, terpinene, limonene, and myrcene) oxygenated terpenes (linalool, geraniol, terpineol, aromatic ethers (myristicin, elemicin, safrole, eugenol, and eugenol)	Matulyte et al. (2020), Suthisamphat et al. (2020)
Myrtaceae	*Syzygium aromaticum* (L.) Merr. & L.M.Perry	Kanplu กานพลู	Flavoring, Thai Mussaman curry	Flower buds	Essential oils, eugenol, eugenyl acetate, β-caryophyllene, glycosides, saponins, flavonoids, hidroxiphenyl propens, hidroxicinamic acids, steroids, tannins, alkaloids, terpenes	Batiha et al., (2020a)
	*Syzygium cumini* L.	Luke wa ลูกหว้า	Drink, dessert, jam	Fruit	Malic acid, oxalic acid, gallic acid, tannins, anthocyanins: cyanidin di-glucosides, delphinidin, petunidin; liquitrigenin, scopoletin, umbelliferon, rosmanol	Abdin et al. (2020), Qamar et al. (2021)
Nymphaeaceae	*Nymphaea pubescens* Willd.	Bua sai บัวสาย	Coconut milk soup or stir-fried	Stem, seeds, flower	Phenolic compounds, flavonoids, gallic acid, shikimic acid	Aimvijarn et al. (2018), Rivas-García et al. (2021)
Pandanaceae	*Pandanus amaryllifolius* Roxb.	Bai tei ใบเตย	Colorant and flavoring	Leaves	Flavonoids, phenolic acids, alkaloids	Reshidan, Abd Muid & Mamikutty (2019)
Pedaliaceae	*Sesamum indicum* L.	Nga งา	Flavoring, decorating, dessert Thai crispy rolls or “Thong Muan”	Seeds	Lignans: sesamin, sesamol, sesaminol, sesamolin; tocopherols, phytosterols, ferulic acid, 5-hydroxy coniferyl alcohol, p-hydroxyphenylacetic acid, other methoxyphenol derivatives	Deme, Narasimhulu & Parthasarathy (2018), Wu et al. (2019)
Phyllanthaceae	*Sauropus androgynus* (L.) Merr.	Phak waan baan ผักหวานบ้าน	Vegetable, boiled, stir-fried, soup	Leaves	Sterols, tannins, saponins, alkaloids, flavonoids, terpenoids, lignan glycosides, phenols, catechol, cardiac glycosides, and acidic compounds	Arif & Shetty (2020)
Piperaceae	*Piper nigrum* L.	Phrik Thai พริกไทย	Black, white, fresh green pepper	Seeds, fruit	Alkaloid: piperine and derivatives isopiperine, chavicine, isochavicine, piperanine, piperettine, piperylin A, piperolein B, pipericine; monoterpenes, sesquiterpenes	Joshi, Shrestha & Adhikari (2018), Tiwari, Mahadik & Gabhe (2020)
	*Piper sarmentosum* Roxb.	Chaplu ชะพลู	Salad wrap “Miang Kham”, curry	Leaves	Elemicin, methoxyeugenol, naringenin, methyl piperate, beta-asarone, brachyamide B, piperitol, guineensine, didymin, quercetin, amurensin, hesperidin, difucol	Junairiah & Zuraidassanaaz (2020)
Plantaginaceae	*Limnophila aromatica* (Lam.) Merr.	Phak khayang ผักแขยง	Raw, soup	Arial part	Essential oil: methyl benzoate, pulegone, limonene, (+)-trans-isolimonene, α-humulene	Dai et al. (2015)
Poaceae	*Cymbopogon citratus* (DC.) Stapf	Ta khrai ตะไคร้	Flavoring, Tom Yum, deep-fried	Stalk	Essential oils: citral, mycrene, genariol, citronellol (cymbopogonol and cymbopogone); α-oxobisabolene, terpenoids (cymbopogonol and cymbopogone); flavonoids	Oladeji et al. (2019)
Polygonaceae	*Persicaria odorata* (Lour.) Soják	Phak paw ผักแพว	Flavoring, fresh, boiled	Leaves	Essential oils: dodecanal, decanal, cis-caryophyllene, alpha-humulene, caryophyllene oxide, humulene epoxide II, drimenol, E-15-Heptadecenal, 3, 7, 11, 15-tetramethyl-2-hexadecen-1-ol; gallic acid, apigenin, ferulic acid, quercetin, ellagic acid, *p*-coumaric acid	Chansiw et al. (2018), Ridzuan & Wan Salleh (2019), Řebíčková et al. (2020)
Rutaceae	*Citrus × aurantium* L.	Som sa ส้มซ่า	Fruit juice and peel are used to flavor crispy rice noodles, curry	Peels blossoms	Essential oils: linalool, limonene Essential oils: linalool, α-terpineol, (*R*)-limonene, linalyl acetate	Azhdarzadeh & Hojjati (2016), Shen et al. (2017)
	*Citrus hystrix* DC.	Makrut มะกรูด	Leaves, juice, and peel is used in curry paste, flavoring, garnish	Peels leaves	Phenolic compounds, flavones, terpenoids, mainly β-pinene, limonene, sabinene, furanocoumarins Phenolic compounds, citronellal, terpenoid agrostophillinol, α/β-pinene, limonene, terpinen-4-ol	Kidarn et al. (2018), Anuchapreeda et al. (2020)
	*Citrus aurantifolia* (Christm.) Swingle	Manao มะนาว	Juice and peel used in flavoring, garnish, beverage	Fruit peels	Monoterpenes like limonene, β-pinene, β-terpinene, citral; alkaloids, carotenoids, coumarins, flavonoids, phenolic acids, triterpenoids Triterpenoid limonoids; sesquiterpenes *p*-caryophyllene, santal-10-en-2-ol; monterpenes limonene, *p*-pinene, geraniol, neral, geranial, citronellal	Jain, Arora & Popli (2020)
	*Zanthoxylum rhetsa* DC.	Ma khwaen มะแขว่น	Fruit and seed used as spice; shoot consumed as vegetable	Fruit, seeds stem, root bark	Sabinene, 4-terpineol, germacrene, gramma-terpinene, alpha-terpinene Alkaloids: isoquinoline and quinolone	Duangyod et al. (2020), Maduka & Ikpa (2021)
Solanaceae	*Capsicum frutescens* L.	Phrik khinu พริกขี้หนู	Garnishing, spicy flavoring	Fruit	Capsaicin, dihydrocapsaicin, capsiconinoids, capsinoids; saponin CAY-1	Batiha et al. (2020b)
	*Capsicum annuum* L.	Phrik chifah พริกชี้ฟ้า	Garnishing, flavoring	Fruit	Capsaicinoids (mainly capsaicin and dihydrocapsaicin); sinapoyl and feruloyl glycosides (red pepper), quercetin-3-O-l-rhamnoside (green pepper)	
Solanaceae	*Lycopersicon esculentum* Mill.	Makhuea thet มะเขือเทศ	An ingredient in papaya salad and spicy salad, soup	Fruit	Carotenoids (lycopene and β-carotenoids), phytosterols (β-sitosterol, campesterol and stigmasterol), phenolic acids (quercetin, kaempferol, narin-genin, lutein, caffeic, ferulic and chlorogenic acids)	Ali et al. (2021)
	*Solanum stramoniifolium* Jacq.	Ma uek มะอึก	Component of Thai chili paste and northeastern papaya salad recipe	Fruit Root	Phenolic compounds Alkaloids, flavonoids, tannins, triterpenes, saponins, solasodine glycoalkaloid (solamargine)	Svobodova et al. (2017)
	*Solanum torvum* Sw.	Makhuea puang มะเขือพวง	Unripe fruit consumed as vegetable and garnish in Thai curries	Fruit Stem	Spirostanol saponins, alkaloids, flavonoids, phenols, tannins, glycosides, tocopherol Steroidal Saponins	Lee et al. (2017b), Darkwah et al. (2020), Lacmago et al. (2021)
Zingiberaceae	*Alpinia galanga* L.	Kah ข่า	Flavoring in curry paste, soup Tom Yum and Tom Kah	Rhizome	Phenolic compounds: ferulic acid, apigenin, vanillic acid, kaempferol, kaempferol-3-O-methylether, luteolin, chrysin, 1’-acetoxyeugenol acetate, 4-hydroxybenzoic acid; terpenoids: galangalditerpene A-B, 1,8-cineole, α-pinene	Khairullah et al. (2020)
	*Boesenbergia rotunda* (L.) Mansf.	Krachai กระชาย	Fish dishes, raw after peeled	Rhizome	Essential oils, flavonoids, polyphenols, chalcone Boesenbergin A, diarylheptanoid panduratin A	Rosdianto et al. (2020), Mohan et al. (2020a), Kanjanasirirat et al. (2020)
	*Curcuma longa* L.	Kamin ขมิ้น	Colorant, flavoring, white turmeric is eaten as a raw vegetable	Rhizome	Polyphenol curcumin, alkaloids, flavonoids, terpenoids	Hewlings & Kalman (2017), Rahaman et al. (2021)
	*Zingiber cassumunar* Roxb.	Plai ไพล	–	Rhizome	Phenylbutenoids, curcuminoids, essential oils, quinines, phenolic compounds, sesquiterpenoids	Han et al. (2021)
Zingiberaceae	*Zingiber officinale* Rosco	Khing ขิง	Flavoring, spice, beverages, and Thai dessert black sesame dumplings in ginger tea	Rhizome	[6]-gingerol and its derivatives, monoterpenes (phellandrene, camphene, cineole, citral, and borneol), sesquiterpenes (zingiberol, zingiberene, zingiberenol, sesquiphellandrene, s-bisabolene)	Karunakaran & Sadanandan (2019)

Many compound classes have been screened and identified in the plant species reviewed here and found to possess various medicinal properties. For example, essential oils or volatile oils, which mainly contain terpenes and terpenoids that are known as antioxidant, antimicrobial, anti-inflammatory, antidiabetic, anticancer, and antiallergic compounds, are found in aromatic plants belonging to the families Apiaceae, Clusiaceae, Lamiaceae, Lauraceae, Myristicaceae, Myrtaceae, Rutaceae, and Zingiberaceae (Marrelli et al., 2020; Masyita et al., 2022). Likewise, the alkaloid piperine and its derivatives are found in plants of the family Piperaceae and have several documented biological activities (Tiwari, Mahadik & Gabhe, 2020). Other major classes of phenolic compounds include polyphenols, phenolic acids, flavonoids, tannins, stilbenes, lignins, lignans, and coumarins, which have shown anti-inflammatory, anti-infective, antiproliferative, and antioxidant activities (Luna-Guevara et al., 2018). Curcuminoids are another group of polyphenols found in *Curcuma* species of the family Zingiberaceae that have been reported as having antioxidant, anti-inflammatory, antimutagenic, antimicrobial, and anticancer properties (Hewlings & Kalman, 2017). Meanwhile, natural xanthones are an important class of compounds in *Garcinia* species of the family Clusiaceae that have potential for treating inflammatory skin diseases (Gunter et al., 2020). Notably, xanthones are also found in the root part of *Cratoxylum formosum* in the family Hyperiaceae (Table 1). Hence, non-edible parts of plants should be evaluated for their potential as additional sources of new bioactive compounds (Svobodova et al., 2017; Rodanant et al., 2017; Maduka & Ikpa, 2021). Among the 69 plant species reviewed here, the commercially used species have been thoroughly investigated for constituent bioactive substances (Jantan et al., 2019; Oyeyinka & Afolayan, 2020; Marefati et al., 2021; Sari, Bellatasie & Ifora, 2021). However, there is very little literature on some less-widely used plant species, such as *Telosma cordata* in the family Apocynaceae (Nguyen, 2020).

Individual plants that are also used in traditional medicine and have shown various pharmacological properties are listed in Table 2. The claimed traditional uses of the selected plants are supported by updated documentation of their pharmacological activities, including antioxidant, antimicrobial, anti-inflammatory, hypolipidemic, antihypertension, antidiabetic, and other like health benefits. In some cases, it is unclear which part of the plant is used to obtain the mentioned therapeutic effect, and there is yet insufficient comparative study of phytochemical and biological activity in different parts of a given plant species. Content of bioactive compounds might vary in different parts of the same plant and in plants of closely related genera (Buathong et al., 2019). Thus, comprehensive studies of the chemical composition and bioactive components in different plant parts and in other genera that have closed taxonomic relationships with the plants listed in this work will open more possibilities for the discovery of new sources and active compounds and hence the expansion of phytomedicine development.

**Table 2:** Traditional and medicinal uses of plants ingredient in Thai food.

Family	Botanical name	Local name	Part	Ethnomedicinal	Pharmacological properties	References
Amaranthaceae	*Suaeda maritima* (L.) Dumort.	Cha kram ชะคราม	Juice leaves	Hepatitis Liver, heart, and lipid disorders	Antiviral, hepatoprotective, anti-inflammatory, antioxidant Hepatoprotective, antioxidant	Nayak et al. (2018), Bilal & Hossain (2019)
Amaryllidaceae	*Allium ascalonicum* L.	Hom daeng หอมแดง	Bulb	Relieve fevers, flatulence, infections	Antibacterial, antivirus, anti-diabetic, antioxidant, anti-inflammation	Ounjaijean et al. (2018)
	*Allium cepa* L.	Hom yai หอมใหญ่	Bulb	Stomach diseases, throat infection, hepatitis, fever, headache, cholera, dysentery, common cold, arthritis	Antioxidant, anticancer, hypolipidemic, antidiabetic, cardioprotective, neuroprotective, antimicrobial anti-inflammatory, antiglycemic	Kothari, Lee & Kim (2020), Marefati et al. (2021)
	*Allium sativum* L.	Kra thiam กระเทียม	Bulb	Common cold, fever, coughs, asthma, wounds, prevention of infectious diseases, including sexually transmitted diseases, tuberculosis, respiratory tract	Antibacterial, anti-inflammatory, antiviral, antitumor, improvement of immunity, antioxidant, anticoagulant, protection of the liver, balancing intestinal microbiota	Hussein, Hameed & Hadi (2017), Shao et al. (2020), Rouf et al. (2020)
Apiaceae	*Apium graveolens* L.	Khuen chai คื่นช่าย	Seeds, leaves, stems	Gout, rheumatism, urinary tract inflammation, arthritis, diuretic for stimulation of the glands, bile, kidney stones, regulate the intestines, increase appetite, prophylaxis for nerve agitation, bronchitis, hepatitis, lower blood pressure, joint problems, libido stimulant, increase breast milk secretion	Antimicrobial, antiparasitic, cardioprotective, gastroprotective, neuroprotective, hypolipidemic, anti-inflammatory, anti-infertility	Salehi et al. (2019b), Emad et al. (2020), Khairullah et al. (2021)
Apiaceae	*Centella asiatica* (L.) Urb.	Bua bok บัวบก	Leaves	Skin, pain, neurological, endocrine, cardiovascular, gastrointestinal, immune, and gynecological diseases, rheumatoid arthritis, ozaena, sore throat, ulcers, burns, leprosy, scrofula	Anti-inflammatory, anti-oxidative stress, anti-apoptotic effects, improvement in mitochondrial function	Sun et al. (2020), Hafiz et al. (2020), Ramli, Xian & Mutalib (2020)
	*Coriandrum sativum* L.	Phak chi ผักชี	Whole plant	Disorders of the digestive, urinary, and respiratory systems, as well as diabetes, inflammation	Antidiabetic, diuretic, cholesterol-lowering, anticancer, anti-inflammatory, antifungal, antihelmintic	Kothalawala et al. (2020), Sari, Bellatasie & Ifora (2021)
	*Cuminum cyminum* L.	Yira ยี่หร่า	Seed	Digestive disorders, chronic diarrhoea, dyspepsia, acute gastritis, diabetes, cancer	Antidiabetic, neuroprotective, cardioprotective, chemo preventive, anti-inflammatory	Srinivasan (2018)
	*Eryngium foetidum* L.	Phak chi farung ผักชีฝรั่ง	Leaves	Diabetes, rheumatism, cold, asthma, cough, sinusitis, stomach disorders	Antioxidant, anti-proliferative, antimicrobial, anti-inflammatory, antidiabetic	Thomas et al. (2017), Prabha, Athoibi & Dsouza (2019)
Apocynaceae	*Telosma* cordata (Burm. f.) Merr.	Khachon ขจร	Flower buds leaves	Conjunctivitis Wound, scaby, ulcer, headache	Antimicrobial, antidiabetic, antioxidant -	Nguyen (2020)
Basellaceae	*Basella alba* L.	Phak plung ผักปลัง	Leaves	Wound healing, androgenic, skin problems, diarrhoea, dysentery, laxative	Anticancer, antiviral, antioxidant, anti-inflammatory, anti-cholesterol, anti-ulcer, antimicrobial, anti-hypoglycemic, antiproliferative	Chaurasiya et al. (2021)
Caricaceae	*Carica papaya* L.	Malako มะละกอ	Fruit, leaves	Dengue fever, diabetes, malaria, fungal infections, skin aging, wound healing, and cancer	Anti-inflammatory, anticancer, antioxidant, antibacterial, and antiviral	Sharma et al. (2020), Haddad et al. (2020), Kong et al. (2021)
Cleomaceae	*Cleome gynandra* L.	Phak Sian ผักเสี้ยน	Leaves seeds root	Ticks and flea prevention, earache, eye wash Anthelmintic, coughing, applied externally for headage, stomach pain Mild reduce fever	Anti-inflammatory, antioxidant, anticancer, immunomodulator, antidiabetic	Adhikari & Paul (2018), Kanimathi et al. (2019)
Clusiaceae	*Garcinia cowa* Roxb. ex Choisy	Cha moung ชะมวง	–	Wounds, ulcers, dysentery	Antioxidant, anti-inflammatory, leishmanicidal, antiprotozoal	Santo et al. (2020)
	*Garcinia schomburgkiana* Pierre	Ma dan มะดัน	–	–	Anti-inflammatory, antibacterial, antioxidant, antitumor, antifungal, anti-HIV	Do et al. (2020)
Cucurbitaceae	*Coccinia grandis* (L.) Voigt	Tum lueng ตำลึง	Leaves	Diabetics, skin diseases, jaundice, biliary disorders, coughs, spleen disorders, respiratory problems, mucus, leprosy, acne, diabetes, mucus in stool, goiter, antidote to poison, scabies, hypertension, abscess, lack of appetite, vomiting, dysentery, burns	Antioxidant, antimicrobial, cytotoxic, antimutagenic, antiulcer, hepatoprotective, expactorants, analgesic, anthelmintic, antidibetic, mast cell-stabilizing, anti-anaphylactic, antihistaminic, anti-inflammatory	Laboni et al. (2017)
	*Cucurbita moschata* Duchesne	Fak Thong ฟักทอง	Seeds pulp peel	Parasitic diseases caused by worms Reduced blood glucose and increased plasma insulin Hepatic disorders, peptic ulcer, gastrointestinal bleeding, wounds, burn	Chemopreventive agent, antimicrobial, antihyperglycemic Antimicrobial, antidiabetic, cardioprotective, hypoglycaemic, antioxidative, anticancer, immunomodulatory, neuroprotective, anti-inflammatory Antioxidant, antibacterial	Shaban & Sahu (2017), Salehi et al. (2019a), Kulczynski & Gramza-Michałowska (2019), Bahramsoltani et al. (2017)
Cucurbitaceae	*Momordica charantia* L.	Mara มะระ	Fruit	Hyperglycemia, oxidative stress, cancer, colitis	Antiulcer, anthelmintic, antidiabetic, anti-inflammatory, antimicrobial, antihyperglycemic, anticancer	Villarreal-La Torre et al. (2020), Ünal et al. (2020)
	*Lagenaria siceraria* (Molina) Standl.	Nam Tao น้ำเต้า	Fruit	Rheumatism, insomnia, diuretic, urinary disorders, excessive thirst, emetic, sedative, purgative, cooling, diuretic, liver disorder, pectora	Antioxidant, laxative, cardioprotective, diuretic, hepatoprotective, hypolipidemic, central nervous system stimulant, anthelmintic, antihypertensive, immunosuppressive analgesic, adaptogen	Tyagi ,Sharma & Shrivastava (2017)
	*Luffa acutangular* (L.) Roxb.	Bob ream บวบเหลี่ยม	Fruit	Jaundice, swollen hemorrhoids, headache	Hepatoprotective, diabetic, hyperlipidemic, CNS depressant, ulcer, cancer, immunomodulatory, antibacterial	Shendge & Belemkar (2018), Panicker (2020)
Dilleniaceae	*Dillenia indica* L.	Ma tad มะตาด	Arial part fruit	Abdominal and joint pain, cough, diarrhoea, fever, tumours, diabetes, toning up the nervous system, removing fatigue Skin inflammation, kidney diseases	Anti-inflammatory, antimicrobial, antidiabetic, hypolipidemic, antidiarrhoeal Anti-leukemic, anticancer antidiarrheal, antioxidant, CNS depressant, anti-inflammatory	Kviecinski et al. (2017), Sen, Chakraborty & Kalita (2018)
Euphorbiaceae	*Phyllanthus emblica* L.	Ma kham pom มะขามป้อม	Fruit	Sore throat, cough, dry mouth, diarrhoea, jaundice, inflammation, diabetes mellitus, constipation, asthma	Immunosuppressive, antioxidant and anti-inflammatory, anti-microbial, hepatoprotective	Jantan et al. (2019), Li et al. (2020)
Fabaceae	*Acacia pennata* (L.) Willd.	Cha om ชะอม	Leaves bark root bark	Body aches, headaches, fevers, helping digestion for infants Asthma and bronchitis Stomach pain, bronchitis, cholera, asthma	Antinociceptive, anti-inflammatory, antifungal, DNA protection for drug abuse harmful effects, anti-flatulent, anti-parasitic	Aye et al. (2019), El-Taher et al. (2021)
	*Clitoria ternatea* L.	Anchan อัญชัน	Various tissue	Enhance cognitive functions, alleviate symptoms of numerous ailments including fever, inflammation, pain, diabetes	Nootropic, anti-convulsant, anti-depressant, anti-anxiety, anti-stress, antioxidant, anti-inflammatory, anti-hyperlipidemic, anti-diabetic, analgesic, cytotoxicity, platelet aggregation inhibitory, hepatoprotective activities	Gollen, Mehla & Gupta (2018), Oguis et al. (2019)
	*Neptunia prostrata* (Lam.) Baill.	Phak kra ched ผักกระเฉด	Leaves	Astringent, sweet, refrigerant, diuretic, antidiarrheal, anti-helmintic, anodyne, dysentery, intestinal infection, fever, earache, poisoning, constipation, gastritis	Antioxidant, α-glucosidase inhibitors, anti-tumor, antibacterial, and α-glucosidase inhibitory, anti-inflammatory, antiulcer, anticancer	Sagolshemcha & Singh (2017), Lee et al. (2019)
	*Leucaena leucocephala* (Lam.) de Wit	Kra tin กระถิน	Leaves	Stomachache, contraceptive, abortifacient agent	Antimicrobial, anticancer, anti-inflammatory, anti-asthma, diuretic, antiarthritic, antifouling, anti-retroviral, anti-diabetic, anti-scabies, antiprotozoal, chemopreventive, immunostimulant, lipoxygenase inhibitor	Zayed, Sallam & Shetta (2018), Zayed, Wu & Sallam (2019)
Fabaceae	*Parkia speciosa* Hassk.	Sataw สะตอ	Seeds and other	Loss of appetite, kidney disorder, diabetes, cardiovascular diseases, headache, severe cough, bronchitis	Anti-hypertensive, antioxidative, anti-inflammatory, anticancer, antimicrobial, antinociceptive	Chhikara et al. (2018), Saleh et al. (2021)
	*Sesbania grandiflora* (L.) Pers.	Dok Kae ดอกแค	Flower leaves	Astringent, fever, catarrh relief, nyctalopia eyes treatment, headache Thrombosis, diarrhea, inflammatory diseases, bronchitis, cough, vomiting, wounds, ulcer, diarrhea, dysentery, catarrh, headache	Anti-inflammatory, antipyretic, antimicrobial, hepatoprotective, anti-plaque, anti-tumor	Mohiuddin (2019), Aye et al. (2019)
	*Senna siamea* (Lam.) H.S.Irwin & Barneby	Khilek ขี้เหล็ก	Leaves	Pain, oedema, constipation, infectious diseases	Laxative, anti-inflammatory analgesic	Ntandou et al. (2018), Nas, Oyeyi & Ali (2018)
	*Tamarindus indica* L.	Ma kham มะขาม	Fruit	Inflammation, stomach pain, throat pain, rheumatism, wound healing, diarrhea, dysentery, parasitic, infestation, fever, malaria, respiratory, helminthes infections, constipation, cell cytotoxicity, gonorrhea, eye diseases, aphrodisiac	Anti-inflammatory, analgesic, antioxidant, hypolipidemic, anti-helminthic, antimicrobial, hepatoprotective, anti-asthmatic, weight-reducing	Komakech et al. (2019), Borquaye et al. (2020)
Gnetaceae	*Gnetum gnemon* L.	Phak lueng ผักเหลียง	Leaves	Malarial related fever, enhance health and stimulate milk production in pregnant women	Antioxidant, antiplasmodial, antibacterial, tyrosinase inhibitory	Dutta et al. (2018), Le et al. (2020)
Hypericaceae	*Cratoxylum formosum* (Jacq.) Benth. & Hook.f. ex Dyer	Phak tiu ผักติ้ว	Leaves	Fevers, diarrhea, itch, ulcer, coughs, stomachache, food poisoning, internal bleeding	Antioxidant, antimicrobial, anti-inflammatory	Rodanant et al. (2017), Juanda et al. (2019)
Lamiaceae	*Mentha × cordifolia* Opiz ex Fresen.	Saranae สะระแหน่	Leaves	–	Analgesic, antioxidant, insecticidal, antimicrobial, antispasmodic, antiplatelet, anti-inflammatory	Sevindik (2018)
	*Ocimum sanctum* L.	Kaphrao กระเพรา	Leaves	Ulcers, skin and mouth infections, fever, coughs, respiratory disorders, heart diseases, enhance memory	Antioxidant, antimicrobial, anti-inflammatory, adaptogen, immunomodulator, antidiabetic, anti-fertility, hepatoprotective, cardioprotective	Kaur et al. (2020), Almatroodi et al. (2020)
	*Ocimum gratissimum* L.	Yira ยี่หร่า	Leaves	Inflammatory bowel diseases, diarrhea, fungal infections, fever, cold catarrh	Anti-colitis, anti-oxidative, anti-inflammatory, antibacterial, antimalarial	Alabi et al. (2018)
	*Ocimum × africanum* Lour.	Maeng lak แมงลัก	Leaves	Cold, fever, parasitic infestations, inflammation of joints, headaches, skin diseases, lowering blood glucose, dysentery, diarrhoea, reduce constipation, lipid peroxidation	Antiarthritic, anti-inflammatory, insecticidal, antimicrobial, antioxidant, anthelmintic, antidiabetic	Marrelli et al. (2020), Chetia et al. (2021)
	*Ocimum basilicum* L.	Horapha โหระพา	Leaves	Kidney disorders, earache, menstrual irregularities, arthritis, anorexia, colds treatment, malaria, fevers, coughs, flu, asthma, bronchitis, influenza, diarrhea	Anti-cancer, radioprotective, antimicrobial, anti-inflammatory, immunomodu-latory, anti-stress, antidiabetic, anti-pyretic, anti-arthritic, antioxidant, prophylactic agent in cardiovascular disease	Shahrajabian, Sun & Cheng (2020)
Lauraceae	*Cinnamomum verum* J.Presl	Ob chei อบเชย	Stem bark	Prevention of nausea and vomiting, common cold, cardiovascular diseases, chronic gastrointestinal disorders, general stimulant, microbial infections, antiseptic	Antioxidant, antimutagenic, antidiabetic, anticancer, antimicrobial, anti-inflammatory	Schink et al. (2018), Parham et al. (2020)
Meliaceae	*Azadirachta indica* A. Juss. var. siamensis Valeton	Sadao สะเดา	Leaves	Cancer, hypertension, heart diseases, diabetes	antipyretic, fungicidal, antihistamine, antiseptic, anti-inflammatory, antioxidant, antimicrobial, anticancer, antidiabetic	Islas et al. (2020)
Menispermaceae	*Cissampelos pareira* L.	Khruea ma noi เครือหมาน้อย	Leaves, root	Ulcer, rheumatism, fever, asthma, cholera, diarrhoea, rabies, blood purifying, snakebite, malaria, pneumonia	Anti-inflammatory, anti-leukemic, antinociceptive, anti-arthritic, anthelmintic, curariform, cardioprotective, antioxidant, immunomodulatory, chemo-modulatory, antibacterial	Iram et al. (2017), Kumari et al. (2021)
	*Tiliacora triandra* Diels	Yanang ย่านาง		Pyretic, bacterial infections, detoxification, immune modulator agent	Antioxidant, anti-inflammatory, hepatic glucose production inhibitory, anticancer, anti-pyretic, acetylcholinesterase inhibitory	Weerawatanakorn et al. (2018), Makinde et al. (2019), Pasachan et al. (2021)
Moringaceae	*Moringa oleifera* Lam.	Ma rum มะรุม	Leaves, seed	Skin infection, asthma, diabetes, diarrhea, arthritis, inflammation, cough, fever, headache, cardiovascular and gastrointestinal diseases	Antioxidant, anti-inflammatory, antiarthritic, antitumor, antimicrobial, hepatoprotective	Jaja-Chimedza et al. (2017), Xu, Chen & Guo (2019), Saleem, Saleem & Akhtar (2020)
Musaceae	*Musa × paradisiaca* L.	Hua Plee หัวปลี	Flower juice	Stimulate breastmilk (Thailand), bronchitis, constipation, ulcers, inflammation of eyes, nervous debilities	Antioxidant, anti-inflammatory, antimicrobial, anti-obesity, pancreatic lipase inhibition, antimalarial	Shubham et al. (2019)
Myristicaceae	*Myristica fragrans* Houtt.	Luke Chan thet ลูกจันทน์เทศ	Seeds	Stomach disorders, peptic ulcer, nausea, dyspepsia, gastrointestinal tract	Antibacterial, anti-inflammatory, antioxidant, anticancer, antiseptic, antiparasitic	Suthisamphat et al. (2020)
Myrtaceae	*Syzygium aromaticum* (L.) Merr. & L.M.Perry	Kanplu กานพลู	Flower	Vomiting, flatulence, nausea; liver, bowel, and stomach disorders; microbial infectious diseases, dentistry	Analgesic, antioxidant, anticancer, antiseptic, anti-depressant, antispasmodic, anti-inflammatory, antiviral, antifungal, and antibacterial	Batiha et al. (2020a)
	*Syzygium cumini* L.	Luke wa ลูกหว้า	Fruit	Cardiometabolic disorders, gastric issues, diabetes, dysentery	Antidiabetic, antihyperglycemic, antihyperlipidemic, anti-inflammatory, cardioprotective, antioxidant	Abdin et al. (2020), Qamar et al. (2021)
Nymphaeaceae	*Nymphaea pubescens* Willd.	Bua sai บัวสาย	Flower	Circulatory system syndrome	Anticancer, antioxidant, neuroprotective	Aimvijarn et al. (2018), Rivas-García et al. (2021)
Pandanaceae	*Pandanus amaryllifolius* Roxb.	Bai tei ใบเตย	Leaves	Energize body, reduce fever, flatulence, diabetes mellitus	Antidiabetic, antioxidant, xanthine oxidase inhibitory,	Shukor et al. (2018), Reshidan, Abd Muid & Mamikutty (2019)
Pedaliaceae	*Sesamum indicum* L.	Nga งา	Seeds	Pain, fever, inflammation, constipation, diuretic, healing burns and wounds	Anti-inflammatory, antiaging, anticancer, antidiabetes, antioxidant, anticancer, antinociceptive, hepatoprotective, antiarthritic antihypertensive, chemoprotective	Deme, Narasimhulu & Parthasarathy (2018), Wu et al. (2019), Afroz et al. (2019)
Phyllanthaceae	*Sauropus androgynus* (L.) Merr.	Phak waan baan ผักหวานบ้าน	Leaves	Body weight reduction, postpartum recovery, enhance lactation in feeding mothers, breast milk production, cholecystosis, diorrhea, oral thrush, nasal ulcers, and yaws; erythrema, measles	Anti-inflammatory, antidiabetic, anti-obesity, lactation inducing activity, anti-obesity, antimicrobial, anticancer, analgesic, antipyretic, aphrodisiac, anti-cholesterol, wound healing	Arif & Shetty (2020), Fikri & Purnama (2020)
Piperaceae	*Piper nigrum* L.	Phrik Thai พริกไทย	Seeds	Rheumatism, diabetes, muscular ache, diuretic, increase salivary secretion, promote digestion, cold, cough, germicidal, blood purifier	Anti-inflammatory, anticancer, antimicrobial, anti-larvicidal, pesticide, anti-Alzheimer’s, antidepressant, bioavailability enhancer, immunomodulatory, anti-allergic, antidiabetic	Joshi, Shrestha & Adhikari (2018), Stojanović-Radić et al. (2019), Tiwari, Mahadik & Gabhe (2020)
	*Piper sarmentosum* Roxb.	Chaplu ชะพลู	Leaves	Headache, relieve muscle weakness and pain; toothache, fungoid dermatitis on feet, coughing asthma, pleurisy, hypertension	Antimicrobial, antioxidant, reduced blood sugar level, neuromuscular blocking activity, anti-plasmodial, reduced blood pressure, anti-atherosclerotic	Bactiar & Fahami (2019), Fauzy et al. (2019), Sundar et al. (2019)
Plantaginaceae	*Limnophila aromatica* (Lam.) Merr.	Phak khayang ผักแขยง	Whole plant	Jaundice, fever, digestion system, vascular dysfunction	Anti-inflammatory, anti-tumor, antioxidant	Dai et al. (2015)
Poaceae	*Cymbopogon citratus* (DC.) Stapf	Ta khrai ตะไคร้	Stalk, leaves	Mosquito repellent in Thailand, analgesic, antipyretic, diuretic	Anti-obesity, antibacterial, antifungal, antinociceptive, antioxidants, antidiarrheal, anti-inflammatory, anti-rheumatic, cardioprotective, restrain platelet composition, cure diabetes, gastrointestinal infections, anxiety, or depression, antimalarial, pneumonia	Oladeji et al. (2019)
Polygonaceae	*Persicaria odorata* (Lour.) Soják	Phak paw ผักแพว	Leaves	Treat flatulence, relieve constipation	Anti-inflammatory, antioxidant, anticancer, anti-hemolytic, antibacterial	Chansiw et al. (2018), Ridzuan & Wan Salleh (2019)
Rutaceae	*Citrus × aurantium* L.	Som sa ส้มซ่า	Blossoms	Cough, phlegm, headache, flatulence	Anti-inflammatory, sedative, anti-anxiety, antidepressant, antibacterial, antifungal,	Shen et al. (2017)
Rutaceae	*Citrus hystrix* DC.	Makrut มะกรูด	Leaves Fruit	Teeth and gum for nourishing dental health Herbal shampoo, inflammatory aliments, fever, headache, bad breath, digestion, flu, sore throats	Anti-inflammatory, neuroprotective, anticancer, antioxidant, antibacterial	Kidarn et al. (2018) Anuchapreeda et al. (2020), Pattarachotanant & Tencomnao (2020)
	*Citrus aurantifolia* (Christm.) Swingle	Manao มะนาว	Fruit	Relieve sore throat and moisten the throat in Thailand, hypertension	Anticancer, antimicrobial, antioxidant, antiulcer, anti-inflammatory, hypolipidemic, antityphoid, hepatoprotective, anti-obesity, cardiovascular, anti-cholinesterase	Jain, Arora & Popli (2020)
	*Zanthoxylum rhetsa* DC.	Ma khwaen มะแขว่น	Fruit, seeds Bark	Toothache, dizzy, bloating, reduce muscle strain, cholera, asthma, bronchitis, heart troubles, piles, relief of hiccup, diarrhea, rheumatism Cardiac, respiratory diseases, tooth infection, stomach infection, rheumatism	Adulticidal, larvicidal, pupicidal, oviposition deterrent, herbicidal, antimalarial, anti-tuberculosis, anti-inflammatory, antiseptic, anticholera Antibacterial, anti-inflammatory, analgesic, anticancer, thrombolytic, photoprotective	Soonwera & Phasomkusolsil (2017), Duangyod et al. (2020), Maduka & Ikpa (2021)
Solanaceae	*Capsicum frutescens* L.	Phrik khinu พริกขี้หนู	Fruit	Arthritis, rheumatism, stomach aches, skin rashes, wound healing, stimulate appetite	Antibacterial, antifungal, insecticidal, anthelmintic, larvicidal, antioxidant, anti-inflammatory	Batiha et al. (2020b)
	*Capsicum annuum* L.	Phrik chifah พริกชี้ฟ้า	Fruit	Analgesic and inflammatory	Antimicrobial, anti-inflammatory, cardiovascular, anti-obesity, immunosuppressant, memory enhancing, antidiabetic	
Solanaceae	*Lycopersicon esculentum* Mill.	Makhuea thet มะเขือเทศ	Fruit	–	Anti-inflammatory, antioxidant, anti-atherosclerotic, anti-obesity, antihypertensive, antiplatelet, and lipid lowering	Mozos et al. (2018)
	*Solanum stramoniifolium* Jacq.	Ma uek มะอึก	Fruit Leaves, root	- Thrush, cold, venereal diseases, inflammation, asthma, arthritis, liver problems, malaria, and cancer	Antibacterial Antibacterial, antioxidant, anti-inflammatory, cytotoxicity	Svobodova et al. (2017)
	*Solanum torvum* Sw.	Makhuea puang มะเขือพวง	Fruit Leaves	Fever, cough, wounds, pain, liver troubles, tooth decay, reproductive problems, arterial hypertension, antidote -	Antioxidant, anti-inflammatory, antimicrobial, antiulcer, cardioprotective, analgesic, nephro-protective Hepatoprotective, immunomodulatory	Darkwah et al. (2020) Innih, Agu & Eze (2018)
Zingiberaceae	*Alpinia galanga* L.	Kah ข่า	Rhizome	Respiratory diseases, stomach disorders, diarrhea, rheumatism, inflammation, diabetes, neurological disorders	Antimicrobial, anti-inflammatory, antioxidant, anti-hepatotoxic, antidiabetic, immunomodulatory, anti-ulcer, antitumor, anti-allergic, anti-HIV	Khairullah et al. (2020)
	*Boesenbergia rotunda* (L.) Mansf.	Krachai กระชาย	Rhizome	It is believed to promote a healthy immune system, alleviating many gastrointestinal disorders	Anti-inflammatory, antimicrobial, anticancer, antimutagenic, antiparasitic, antiulcer, antileukemia, hepatoprotective, anti-SARS-CoV-2	Rosdianto et al. (2020), Kanjanasirirat et al. (2020), Mohan et al. (2020a)
	*Curcuma longa* L.	Kamin ขมิ้น	Rhizome	Body mask (Thai folk), stomach ulcers, skin diseases, chest pain, cough, diabetes, rheumatism	Antioxidant, anti-inflammatory, antimutagenic, antimicrobial, anticancer	Hewlings & Kalman (2017), Rahaman et al. (2021)
Zingiberaceae	*Zingiber cassumunar* Roxb.	Plai ไพล	Rhizome	Relieve flatulence and diarrhea, combined with other herbs as an herbal ball to alleviate muscle and musculoskeletal pain, inflammation, bruise, sprain, and strain	Antimicrobial, anticancer, anti-inflammatory, neuroprotective, antioxidant, anti-aging, skin-whitening, analgesic, antipyretic	Chongmelaxme et al. (2017), Li et al. (2019), Han et al. (2021)
	*Zingiber officinale* Rosco	Khing ขิง	Rhizome	Cold, headache, coughs, aphthous ulcers, nausea, colic, heart palpitations, stimulates appetite, digestion-stimulation, rheumatism	Anti-inflammatory, antiviral, radioprotective, antioxidant, anticancer, antidiabetic, nephroprotective, hepatoprotective, larvicidal, analgesic, immunomodulatory	Mutthuraj et al. (2020), Dissanayake, Waliwita & Liyanage (2020)

## An updated list of bioactivities of the selected plants

Inflammation is divided into two types: acute and chronic. Acute inflammation is a defense mechanism induced in response to harmful stimuli such as infections, injuries, and chemicals and is recognized by swelling, redness, fever, and pain. Chronic inflammation will occur if acute inflammation lingers for a long time, eventually leading to oxidative stress-mediated diseases, heart disease, diabetes, cancer, arthritis, and bowel diseases like ulcerative colitis (Marefati et al., 2021). Infectious diseases caused by microorganisms and pathogenic viruses can also induce cellular responses, activating signaling molecules such as NO and ROS that contribute to the inflammatory process by triggering the release of a cytokine storm that includes pro-inflammatory cytokines like TNF-α and interleukin (IL-1β, IL-6); this results in cell death (apoptosis) that can lead to acute respiratory distress or syndrome (ARDS), as seen after infection with SARS-CoV-2 (Donma & Donma, 2020). In this regard, we provide a recent backdated review to update the field on the discovery of many extracts and isolated compounds from plant species, with a specific focus on anti-inflammatory properties observed upon administration in study models and on antimicrobial effects demonstrated against antibiotic-resistant bacteria and clinically significant viruses.

### Anti-inflammatory activity

Many researchers and scientists have attempted to demonstrate the potential of selected plants as bioactive anti-inflammatory agents; this activity is assessed by decreasing and inhibiting different mediators and pathways, and by microscopic changes. Among the selected species, a total of 49 species in 24 families have recently been investigated for their anti-inflammatory effects through *in vitro* and *in vivo* experimental studies (Table 3). Crude extracts, fractions, juice, and isolated compounds of Thai food plant ingredients have all demonstrated the potential to complement searches for anti-inflammatory drugs. For example, supplementation with water-soluble polysaccharide from *Allium sativum* (Apiaceae) can attenuate dextran sulfate sodium-induced colitis in mice (Shao et al., 2020). Daily oral supplementation with anthocyanin extract from *Clitoria ternatea* (Fabaceae) for 1 week effectively reduced carrageenan-induced paw edema in rats as compared with ibuprofen (Yanti et al., 2020). Similarly, administration of a polyphenol-rich extract from *Occimum gratissimum* (Apiaceae) promoted repair of colonic mucosa injuries in a dosage-dependent manner in experimental colitis rats (Alabi et al., 2018).

**Table 3:** Plants that show related anti-inflammatory activities.

Family	Botanical name	Local name	Tested samples	Assay/Study model	Results and mechanisms	References
Amaryllidaceae	*Allium ascalonicum*	Hom daeng หอมแดง	Juice	Lipopolysaccharide (LPS)-stimulated on human vascular endothelial cells	Significantly attenuated the level of IL-6 released	Ounjaijean et al. (2018)
	*Allium sativum*	Kra thiam กระเทียม	Water-soluble polysaccharide extract	Dextran sulfate sodium (DSS)-induced colitis in mice	Reduce DSS-induced colitis by improving mucosal barriers, blocking proinflammatory cytokines IL-6, TNF-α, and IL-1β, and modulating gut microbiota in colitis mice	Shao et al. (2020)
Apiaceae	*Apium graveolens*	Khuen chai คื่นช่าย	Methanol root extract	Acetaminophen-induced hepato-toxicity (AAH) rats.	Lowered serum levels of AST, ALT, ALP, TNF-α, and IL-1β	Emad et al. (2020)
	*Centella asiatica*	Bua bok บัวบก	Ethanolic raw extract	*In vitro* LPS-stimulated RAW 264.7 cells and *in vivo* Sprague Dawley rats	Suppressed the level of pro-inflammatory cytokine/mediators and oxidative stress and consistent to the LPS-induced neuroinflammation Sprague Dawley rats’ model.	Hafiz et al. (2020)
	*Coriandrum sativum*	Phak chi ผักชี	Concoction with *Coscinium fenestratum* (Gaertn.) Colebr.	Carrageenan-induced rat paw-edema	Potent *in vivo* anti-inflammatory activity, significant reduction of ROS, and NO production by rat peritoneal cells and lack of iNOS expression confirmed the low NO production	Kothalawala et al. (2020)
	*Cuminum cyminum*	Yira ยี่หร่า	Isolated atypical nitrogen-containing flavonoid	LPS-stimulated RAW 264.7 cells	Exhibited inhibitory effect on nitride oxide, with IC_50_ of 5.25 μM	Kang et al. (2019)
	*Eryngium foetidum*	Phak chi farung ผักชีฝรั่ง	Hydro-methanolic extract	Heat-induced denaturation of protein and red blood cell (RBC) membrane stabilization	Inhibited heat induced protein denaturation and RBC membrane stabilization at different concentrations	Prabha, Athoibi & Dsouza (2019)
Basellaceae	*Basella alba*	Phak plung ผักปลัง	Aqueous leaf extract	Streptozotocin-induced diabetic rats	Stimulates the recovery of beta-islet morphology by modulating the peripheral production of inflammatory cytokines.	Arokoyo et al. (2018)
Caricaceae	*Carica papaya*	Malako มะละกอ	Freeze-dried leaf juice	AG129 mice infected with DEN-2 dengue virus	The inflammatory cytokine genes: CCL6/MRP-1, CCL8/MCP-2, CCL12/MCP-5, CCL17/TARC, IL1R1, IL1RN/IL1Ra, NAMPT/PBEF1 and PF4/CXCL4 were downregulated.	Norahmad et al. (2019)
Cucurbitaceae	*Coccinia grandis*	Tum lueng ตำลึง	Methanolic leaf extract	*In vitro* scratch wound healing of human keratinocyte and fibroblast	Increased the wound healing process from its antioxidant capacity that acted as a proton donor to neutralize reactive oxygen species and protected the human fibroblasts and keratinocytes from hydrogen peroxide-induced oxidative stress by increasing cell survival rate.	Namchaiw et al. (2021)
	*Cucurbita moschata*	Fak Thong ฟักทอง	Tendrils extract Oleic acid ester of hydroxy oleic acid	NLRP3 inflammasome activation in murine macrophages and human trophoblast cell LPS-stimulated dendritic cells	Attenuated NLRP3 inflammasome activation and suppressed IL-1β secretion dose-dependently, without affecting IL-6 secretion. Moreover, it inhibited NLRP3-dependent pyroptosis in LPS-primed bone marrow-derived macrophages and significantly inhibited IL-1β secretion and pyroptotic cell death in human trophoblast cells. Reduced inflammation in adipose tissue by inhibiting the expression of cytokines IL-12, IL-1β, and TNF-α.	Park et al. (2020), Dong et al. (2021)
	*Momordica charantia*	Mara มะระ	Crude extract	2,4,6-trinitrobenzene sulfonic acid (TNBS)-induced colitis in rat	Showed protective anti-inflammatory effect	Ünal et al. (2020)
	*Luffa acutangular*	Bob ream บวบเหลี่ยม	Ethanolic fruit extract	Carrageenan-induced paw edema in Wistar albino rats	Exhibited moderate anti-inflammatory activity	Palash et al. (2017)
Dilleniaceae	*Dillenia indica*	Ma tad มะตาด	Ethyl acetate fruit extract	Ultraviolet radiation-induced psoriasis-like wounds in rats	Healing effect within 16 days after wound induction which betulinic acid may be an active constituent	Kviecinski et al. (2017)
Euphorbiaceae	*Phyllanthus emblica*	Ma kham pom มะขามป้อม	Ethanolic fruit extract	LPS-induced RAW 264.7 cells	Exhibited antioxidant activity and decreased releasing of pro-inflammatory mediators	Li et al. (2020)
Fabaceae	*Clitoria ternatea*	Anchan อัญชัน	Anthocyanin extract	Carrageenan-induced rat paw edema	Protective activity *via* down-regulating genes of phosphoinositide 3-kinase signaling pathway	Yanti et al. (2020)
	*Leucaena leucocephala*	Kra tin กระถิน	Leaf extract	Oxidized low density lipoprotein (oxLDL) and glucose-induce oxidative stress in human umbilical vein	Reduced oxidative stress condition in impaired fasting blood glucose patients that induce vascular endothelial dysfunction in diabetic and hyperlipidemia	Chatchanayuenyong, Sujayanont & Vuttivirojana (2018)
	*Parkia speciosa*	Sataw สะตอ	Ethyl acetate fraction of empty pod extract	Tumor necrosis factor-α (TNF-α)-induced inflammation human umbilical vein endothelial cells and H9c2 cardiomyocytes	Exhibited anti-inflammatory properties by modulating the NFκB and p38 MAPK pathways	Sevindik (2018), Gui et al. (2019)
	*Sesbania grandiflora*	Dok Kae ดอกแค	Leaf methanolic extract	Carrageenan-induced and formalin-induced rat paw edema	Inhibited formalin and carrageenan induced paw edema in 1–2 h of induction	Karale et al. (2018)
	*Senna siamea*	Khilek ขี้เหล็ก	Aqueous leaf extract	Carrageenan-induced rat paw edema and granuloma cotton pellet	Significant inhibition activity against acute and chronic inflammation. Moreover, the extract also significantly decreases the latency of the first fecal excretion increased the fecal excretion rate for laxative effects.	Ntandou et al. (2018)
	*Tamarindus indica*	Ma kham มะขาม	Fruit pulp extract Root and stem bark extracts	LPS stimulated RAW 264.7 macrophages Carrageenan-induced paw edema in chicks	Inhibit the production of nitric oxide and the iNOS gene expression Exhibited dose-dependent reduction of inflammation	Leya & Anitha (2019) Borquaye et al. (2020)
Gnetaceae	*Gnetum gnemon*	Phak lueng ผักเหลียง	Seed extract	*In vivo* C57BL/6J mice fed with high-fat diet with 1% extract added	Induces brown adipose tissue (BAT) thermogenesis and reduces obesity-associated adipose tissue inflammation, hepatic steatosis, and insulin resistance	Yoneshiro et al. (2018)
Hypericaceae	*Cratoxylum formosum*	Phak tiu ผักติ้ว	Isolated lupeol Leaves crude extract	LPS-stimulated phorbol-12-myristate-13-acetate (PMA)-stimulated-monocytes L-arginine methyl ester hydrochloride treated rats	Inhibited the expression of TNF-α. Significantly alleviated left ventricular hypertrophy associated with reducing oxidative stress markers	Rodanant et al. (2017), Potue et al. (2020)
Lamiaceae	*Ocimum gratissimum*	Yira ยี่หร่า	Polyphenol-rich extract	DSS-induced rat colitis	Showed potential to repair colonic mucosa injury in experimental colitis by regulating pro-inflammatory cytokines production and oxidative stress	Alabi et al. (2018)
	*Ocimum basilicum*	Horapha โหระพา	Leaf extracts	Co-culture of 3T3-L1 Adipocytes and RAW264.7 Macrophages	Anti-inflammatory effect against adipocyte-induced inflammation, possibly through suppression of inflammatory signaling Tnfrsf9 expression (a member of the TNF super-family) as same as reduced expression of inflammatory cytokine mRNA on a co-culture	Takeuchi et al. (2020)
Lauraceae	*Cinnamomum verum*	Ob chei อบเชย	Bark extract, *p*-cymene, *trans*-cinnamal-dehyde	LPS-stimulated THP-1 monocyte-macrophage cell line	Mitigated the phosphorylation of Akt and IκBα. Moreover, *trans*-cinnamaldehyde and *p*-cymene significantly reduced the LPS-dependent IL-8 secretion in THP-1 monocytes.	Schink et al. (2018)
Meliaceae	*Azadirachta indica*	Sadao สะเดา	Leaf extract	Cigarette smoke and LPS-induced pulmonary inflammation in lungs mice	Decreased the production of ROS and the activity of neutrophil elastase in bronchoalveolar lavage fluid, blocking proinflammatory cytokines and the activation of extracellular signal-regulated kinase (ERK) and c-Jun N-terminal kinase (JNK), and other pathways	Lee et al. (2017a)
Menispermaceae	*Cissampelos pareira*	Khruea ma noi เครือหมาน้อย	Pectin	LPS-stimulated RAW264.7 macrophages	DPPH and NO scavenging, NO production is inversely correlated to pectin concentration	Wisidsri & Thungmungmee (2019)
	*Tiliacora triandra*	Yanang ย่านาง	Lyophilized leaf juice	LPS-stimulated RAW264.7 macrophages	Down-regulated the induction of inflammatory iNOS and COX-2 proteins	Weerawatanakorn et al. (2018)
Moringaceae	*Moringa oleifera*	Ma rum มะรุม	Leaf extract	Wistar rats by formaldehyde induced arthritis	Significant antioxidant and anti-arthritic potential by *in vitro* anti-inflammatory assays such as protein denaturation, membrane stabilization and anti-proteinase activities	Saleem, Saleem & Akhtar (2020)
Myristicaceae	*Myristica fragrans*	Luke Chan thet ลูกจันทน์เทศ	Ethanolic aril extract	LPS-induced RAW264.7 cell line	Inhibited NO release and has cytotoxic activity against gastric cancer cell lines (Kato III)	Suthisamphat et al. (2020)
Myrtaceae	*Syzygium aromaticum*	Kanplu กานพลู	Formulated essential oil in absorption base ointment with added oleic and propylene glycol	Croton oil-induced inflammatory mice	Treatment showed less COX-2 expression, number of inflammatory cells, and epidermal thickness	Sugihartini et al. (2019)
Myrtaceae	*Syzygium cumini*	Luke wa ลูกหว้า	Isolated malvidin 3,5-diglucoside	LPS-induced RAW264.7 macrophages	Inhibited NO release and pro-inflammatory mediators like mouse IL-6, IL-1β) and TNF-α	Abdin et al. (2020)
Pandanaceae	*Pandanus amaryllifolius*	Bai tei ใบเตย	Leaf aqueous extract	Fructose-induced metabolic syndrome rat	Improved obesity parameters with neutral effects on inflammatory biomarkers	Reshidan, Abd Muid & Mamikutty (2019)
Pedaliaceae	*Sesamum indicum*	Nga งา	Methoxy phenol compounds in aqueous extract of sesame oil	LPS-induced monocyte-derived macrophages and RAW 264.7 cells	Attenuates inflammatory cytokines	Deme, Narasimhulu & Parthasarathy (2018)
Phyllanthaceae	*Sauropus androgynus*	Phak waan baan ผักหวานบ้าน	Leaf patch	Carrageenan-induced rats	At 400 mg/kg showed healing activity after patch application.	Desnita et al. (2018)
Piperaceae	*Piper nigrum*	Phrik Thai พริกไทย	Green pepper ethanolic extract	LPS-induced RAW 264.7 cells	Significantly suppressed nitrite production and inducible NO synthase expression without being cytotoxic. It also suppressed the LPS-induced phosphorylation of mitogen-activated protein kinases.	Kim et al. (2020)
	*Piper sarmentosum*	Chaplu ชะพลู	Aqueous leaf extract	TNF-α-treated human umbilical vein endothelial cells	Promotes endothelial NO production by stimulating DDAH activity and reducing asymmetric dimethylarginine (ADMA) level, an endogenous inhibitor of endothelial nitric oxide synthase	Sundar et al. (2019)
Rutaceae	*Citrus × aurantium*	Som sa ส้มซ่า	Essential oils from the flower	LPS-induced RAW 264.7 cells	Significant anti-inflammatory activities by inhibiting production of NO, IL6, TNF-α, IL-1β and decreased COX-2 gene and protein expression levels, inhibited NF-κB	Shen et al. (2017)
	*Citrus hystrix*	Makrut มะกรูด	Isolated furanocoumarins	LPS-interferon gamma-induced cell lines	Inhibited NO and iNOS in RAW264.7 cells and COX-2 production in HT-29 and HCT116 cells.	Kidarn et al. (2018)
Rutaceae	*Citrus hystrix*	Makrut มะกรูด	Isolated terpenoid agrostophillinol Ethanolic leaf extract, lupeol	Leukemic and RAW264.7 cells LPS-stimulated and NLRP3 adenosine triphosphate-induced macrophages	Showed anti-leukemic and anti-inflammatory by significantly inhibited IL-6 secretion Significantly reduced the release of pro-inflammatory cytokines and suppressed the expression of both inflammasome genes and NF-κB proteins and NLRP3 signaling pathways	Anuchapreeda et al. (2020), Buakaew et al. (2021)
	*Citrus aurantifolia*	Manao มะนาว	Peel extract	Balb/c mice infected by *Salmonella typhi*	Decreased serum level of IL6	Kasim et al. (2020)
	*Zanthoxylum rhetsa*	Ma khwaen มะแขว่น	Pericarp and seed essential oil extracts	LPS-induced RAW 264.7 macrophages	Showed inhibition of inflammatory mediators (NO, TNF-α, and PGE2)	Imphat et al. (2021)
Solanaceae	*Solanum stramoniifolium*	Ma uek มะอึก	Root extract	LPS-induced RAW 264.7 macrophages	Inhibited NO production	Svobodova et al. (2017)
	*Solanum torvum*	Makhuea puang มะเขือพวง	Isolated spirostanol derivatives	RBL-2H3 basophilic leukemia cell line	Showed anti-metastatic and anti-inflammatory effects against interleukin-4 (IL-4) release in inflammation-associated tumors	Lee et al. (2017b)
Zingiberaceae	*Boesenbergia rotunda*	Krachai กระชาย	Isolated boesenbergin A	Ethanol-induced gastric ulcer *in vivo*	Reduced ulcerated and haemorrhagic areas by boosting gastric mucus production, suppressed inflammatory mediators (TNF-α and IL-6 cytokines) and modulated the oxidative stress response	Mohan et al. (2020a)
	*Curcuma longa*	Kamin ขมิ้น	Co-treatment with *Allium hookeri* extract	Carrageenan-induced air pouch and LPS-induced RAW 264.7 cells	Ratio as 3:7 suppressed inflammatory cytokines and inhibited NF-κB/COX-2/iNOS pathway	Lee et al. (2020)
Zingiberaceae	*Zingiber cassumunar*	Plai ไพล	Gel containing Plai oil–encapsulated niosomes (*E*)-3-(3,4-dimethoxyphenyl)-2-propenal; 1-feruloyloxy cinnamic acid; (1*E*,4*E*,6*E*)-1,7-bis(4-hydroxyphenyl)-1,4,6-heptatrien-3-one; bisdemethoxycurcumin; and curcumin	Inflamed subcutaneous Wistar rat skin by therapeutic ultrasound LPS-induced mouse macrophages cell (RAW264.7)	Decreased skin temperature and blood flow to the lowest level compared to the application of neurofen drug Five active compounds showed anti-inflammatory potentials with NO generation inhibition	Leelarungrayub, Manorsoi & Manorsoi (2017) Li et al. (2019)
	*Zingiber officinale*	Khing ขิง	Essential oil extract	Administering with fresh and dry ginger essential oil (external apply) on Arthritis patients	Decreased rheumatoid arthritis factor and level of c-reactive protein produced	Mutthuraj et al. (2020)

Additionally, plant parts besides the edible ones have shown anti-inflammatory properties, such as with an ethyl acetate fraction of empty pod extract of *Parkia speciosa* (Fabaceae) (Sevindik, 2018; Gui et al., 2019), root and stem bark extract of *Tamarindus indica* (Fabaceae) (Borquaye et al., 2020), and flower essential oil of *Citrus aurantium* (Rutaceae) (Shen et al., 2017). Some bioactive candidates for anti-inflammatory effect have lately been identified, for example: an atypical nitrogen-containing flavonoids from *Cuminum cyminum* (Apiaceae) (Kang et al., 2019); oleic acid ester of hydroxy oleic acid from *Cucurbita moschata* (Cucurbitaceae) (Dong et al., 2021); lupeol from *Cratoxylum formosum* (Hypericaceae) (Rodanant et al., 2017) and *Citrus hystrix* (Rutaceae) (Buakaew et al., 2021); the terpenoid agrostophillinol (Anuchapreeda et al., 2020) and furanocoumarins (Kidarn et al., 2018) also from *Citrus hystrix* (Rutaceae); *p*-cymene and *trans*-cinnamaldehyde from *Cinnamomum verum* (Lauraceae) (Schink et al., 2018); pectin from *Cissampelos pareira* (Menispermaceae) (Wisidsri & Thungmungmee, 2019); malvidin 3,5-diglucoside from *Syzygium cumini* (Myrtaceae) (Abdin et al., 2020); methoxy phenol compounds from *Sesamum indicum* (Pedaliaceae) (Deme, Narasimhulu & Parthasarathy, 2018); spirostanol derivatives from *Solanum torvum* (Solanaceae) (Lee et al., 2017b); boesenbergin A from *Boesenbergia rotunda* (Zingiberaceae) (Mohan et al., 2020a), and the compounds (*E*)-3-(3,4-dimethoxyphenyl)-2-propenal, 1-feruloyloxy cinnamic acid, (1*E*,4*E*,6*E*)-1,7-bis(4-hydroxyphenyl)-1,4,6-heptatrien-3-one, bisdemethoxycurcumin, and curcumin from *Z. cassumunar* (Li et al., 2019), also of the family Zingiberaceae.

Reports of bioactivity have also been published for gel, ointment, and patch formulations, and for co-treatment with selected plants (Table 3). In one case, a concoction of *Coriandrum sativum* (Apiaceae) with *Coscinium fenestratum* showed potent *in vivo* anti-inflammatory activity (Kothalawala et al., 2020). Anti-inflammatory effects with potential in product development have also been exhibited by essential oil from *Syzygium aromaticum* (Myrtaceae) formulated in an absorption base ointment with added oleic and propylene glycol (Sugihartini et al., 2019), a leaf patch from *Sauropus androgynus* (Phyllanthaceae) (Desnita et al., 2018), and a gel containing encapsulated niosomes of Plai oil (*Z. cassumunar*, Zingiberaceae) (Leelarungrayub, Manorsoi & Manorsoi, 2017). Finally, co-treatment using *Curcuma longa* (Zingiberaceae) and *Allium hookeri* extracts at a ratio of 3:7 showed optimal anti-inflammatory properties, indicating a synergistic plant-plant combination effect (Lee et al., 2020).

However, there still remains a need to establish a direct link between a plant extract and its putative bioactive compounds, for example flavonoids and essential oils, to further elucidate the anti-inflammatory role and help design clinical research and address the current insufficient body of evidence. This will allow developing a better understanding and implementation that might promote health maintenance and prevent numerous health conditions and diseases related to the inflammatory response.

### Antibacterial

*Staphylococcus aureus*, *Escherichia coli*, *Pseudomonas aeruginosa*, and *Klebsiella pneumoniae* have been listed as antibiotic-resistant bacteria by the World Health Organization (WHO) since 2017. Of those, *P. aeruginosa* (carbapenem-resistant) and Enterobacteriaceae like *K. pneumoniae* and *E. coli* (carbapenem-resistant) can produce extended-spectrum beta-lactamases (ESBLs) to interfere with beta-lactam antibiotics, and are accordingly ranked as of the utmost priority, followed by *S. aureus* (methicillin-resistant and vancomycin-intermediate and -resistant) (Magryś, Olender & Tchórzewska, 2021). Moreover, as noted in a previous systematic review article, these bacteria have been ranked as the top four targeted pathogenic bacteria for a decade, indicating the ongoing need to combat their antibiotic-resistant strains (Chassagne et al., 2021). The authors also pointed out in that review that among the 15 most represented plant families, those most reported as having antibacterial activities are Lamiaceae, Fabaceae, and Asteraceae.

Here, we provide recent scientific articles on plants used in Thai food, covering a total of 51 plant species in 25 families that have been reported as having antibacterial effects against the abovementioned human pathogenic bacteria (Table 4). In short, several studies have demonstrated broad antibacterial activity against both Gram-positive and Gram-negative bacteria in plant genera belonging to the families of Amaranthaceae (Nayak et al., 2018; Bilal & Hossain, 2019), Amaryllidaceae (Anyaegbunam et al., 2019; Enejiyon et al., 2020; Magryś, Olender & Tchórzewska, 2021), Apiaceae (Salehi et al., 2019b; Ali & Malik, 2020; Aboody, 2021), Cleomaceae (Ganesh, Muthusamy & Jaganathan, 2018; Kanimathi et al., 2019), Fabaceae (Anantaworasakul et al., 2017; Ghasemzadeh et al., 2018; Nas, Oyeyi & Ali, 2018; Noviany et al., 2020; Muhialdin, Abdul Rani & Hussin, 2020), Lamiaceae (Mittal, Kumar & Chahal, 2018; Jesuwenu & Michael, 2017; Melo et al., 2019), Menispermaceae (Uthpala & Raveesha, 2019), Myristicaceae (Dzotam et al., 2018; Kiarsi et al., 2020), Myrtaceae (Moemenbellah-Fard et al., 2020), Poaceae (Subramaniam, Yew & Sivasamugham, 2020), Rutaceae (Sreepian et al., 2019; Srifuengfung et al., 2020), Solanaceae (Obiang et al., 2019), and Zingiberaceae (Beristain-Bauza et al., 2019).

**Table 4:** Plants that show antibacterial activities against some human pathogenic bacteria: *Staphylococcus aureus*, *Escherichia coli*, *Pseudomonas aeruginosa*, *Klebsiella pneumoniae*.

Family	Botanical name	Local name	*S. aureus*	*E. coli*	*P. aeruginosa*	*K. pneumoniae*	References
Amaranthaceae	*Suaeda maritima*	Cha kram ชะคราม	*+*	+	+	+	Nayak et al. (2018), Bilal & Hossain (2019)
Amaryllidaceae	*Allium cepa*	Hom yai หอมใหญ่	*+*	+	+	+	Anyaegbunam et al. (2019), Enejiyon et al. (2020)
	*Allium sativum*	Kra thiam กระเทียม	*+*	+	+	+	Magryś, Olender & Tchórzewska (2021)
Apiaceae	*Apium graveolens*	Khuen chai คื่นช่าย	*+*	NA	+	+	Salehi et al. (2019b), Aboody (2021)
	*Centella asiatica*	Bua bok บัวบก	*+*	+	+	+	Soyingbe, Mongalo & Makhafola (2018)
	*Coriandrum sativum*	Phak chi ผักชี	*+*	+	+	NA	Ali & Malik (2020)
	*Cuminum cyminum*	Yira ยี่หร่า	*+*	+	NA	NA	Wongkattiya et al. (2019)
	*Eryngium foetidum*	Phak chi farung ผักชีฝรั่ง	*+*	+	NA	NA	Dalukdeniya & Rathnayaka (2017)
Basellaceae	*Basella alba*	Phak plung ผักปลัง	*+*	+	NA	NA	Deka et al. (2017)
Caricaceae	*Carica papaya*	Malako มะละกอ	*+*	+	+	NA	Ukaegbu-Obi, Anyaegbunam & Enya (2018)
Cleomaceae	*Cleome gynandra*	Phak Sian ผักเสี้ยน	*+*	+	*+*	+	Ganesh, Muthusamy & Jaganathan (2018), Kanimathi et al. (2019)
Cucurbitaceae	*Coccinia grandis*	Tum lueng ตำลึง	*+*	+	+	NA	Laboni et al. (2017)
	*Cucurbita moschata*	Fak Thong ฟักทอง	–	NA	+	+	Dash & Ghosh (2018)
	*Momordica charantia*	Mara มะระ	*+*	+	+	NA	Villarreal-La Torre et al. (2020)
	*Lagenaria siceraria*	Nam Tao น้ำเต้า	–	NA	+	+	Ahmed & Ashiq (2018), Dash & Ghosh (2018)
Dilleniaceae	*Dillenia indica*	Ma tad มะตาด	*+*	+	NA	NA	Meeprathom, Jongrattanavit & Kooprasertying (2018)
Euphorbiaceae	*Phyllanthus emblica*	Ma kham pom มะขามป้อม	*+*	+	NA	+	Ashalatha, Hemalatha & Raveesha (2019)
Fabaceae	*Clitoria ternatea*	Anchan อัญชัน	*+*	+	NA	NA	Mahmad et al. (2018)
	*Neptunia prostrata*	Phak kra ched ผักกระเฉด	*+*	NA	NA	NA	Chakraverty et al. (2019)
	*Leucaena leucocephala*	Kra tin กระถิน	*+*	NA	NA	+	Umaru, Samling & Umaru (2018)
	*Parkia speciosa*	Sataw สะตอ	*+*	+	+	NA	Ghasemzadeh et al. (2018), Muhialdin, Abdul Rani & Hussin (2020)
	*Sesbania grandiflora*	Dok Kae ดอกแค	*+*	+	*+*	+	Anantaworasakul et al. (2017), Noviany et al. (2020)
	*Senna siamea*	Khilek ขี้เหล็ก	NA	+	+	+	Nas, Oyeyi & Ali (2018)
	*Tamarindus indica*	Ma kham มะขาม	*+*	+	NA	NA	Adeniyi et al. (2017)
Gnetaceae	*Gnetum gnemon*	Phak lueng ผักเหลียง	*+*	+	+	NA	Parhusip et al. (2019), Dayoh & Rahayu (2021)
Lamiaceae	*Ocimum sanctum*	Kaphrao กระเพรา	*+*	NA	NA	+	Mittal, Kumar & Chahal (2018)
	*Ocimum gratissimum*	Yira ยี่หร่า	*+*	+	NA	+	Jesuwenu & Michael (2017), Melo et al. (2019)
	*Ocimum × africanum*	Maeng lak แมงลัก	*+*	+	NA	NA	Chetia et al. (2021)
	*Ocimum basilicum*	Horapha โหระพา	NA	+	NA	NA	Juliet et al. (2019)
Lauraceae	*Cinnamomum verum*	Ob chei อบเชย	*+*	+	+	NA	Kwak, Kim & Kim (2017), Parham et al. (2020)
Meliaceae	*Azadirachta indica*	Sadao สะเดา	*+*	+	+	NA	Maleki et al. (2018)
Menispermaceae	*Cissampelos pareira*	Khruea ma noi เครือหมาน้อย	*+*	+	+	+	Uthpala & Raveesha (2019)
	*Tiliacora triandra*	Yanang ย่านาง	*+*	+	NA	NA	Makinde et al. (2019)
Moringaceae	*Moringa oleifera*	Ma rum มะรุม	*+*	+	+	+	Bancessi et al. (2020)
Myristicaceae	*Myristica fragrans*	Luke Chan thet ลูกจันทน์เทศ	*+*	+	+	+	Dzotam et al. (2018), Kiarsi et al. (2020)
Myrtaceae	*Syzygium aromaticum*	Kanplu กานพลู	*+*	+	+	+	Moemenbellah-Fard et al. (2020)
	*Syzygium cumini*	Luke wa ลูกหว้า	*+*	+	+	NA	Sharma et al. (2017)
Phyllanthaceae	*Sauropus androgynus*	Phak waan baan ผักหวานบ้าน	*+*	+	+	NA	Kuttinath, Haritha & Rammohan (2019)
Piperaceae	*Piper nigrum*	Phrik Thai พริกไทย	+	+	NA	+	Hikal, 2018; Reshmi & Raj (2020)
	*Piper sarmentosum*	Chaplu ชะพลู	*+*	NA	*+*	*+*	Ibrahim & Nazir (2019)
Poaceae	*Cymbopogon citratus*	Ta khrai ตะไคร้	*+*	+	+	+	Subramaniam, Yew & Sivasamugham (2020)
Polygonaceae	*Persicaria odorata*	Phak paw ผักแพว	+	–	–	NA	Chansiw et al. (2018)
Rutaceae	*Citrus hystrix*	Makrut มะกรูด	*+*	+	+	+	Sreepian et al. (2019), Srifuengfung et al. (2020)
	*Citrus aurantifolia*	Manao มะนาว	NA	+	NA	+	Zage, Tajo & Ali (2018)
Solanaceae	*Lycopersicon esculentum*	Makhuea thet มะเขือเทศ	*+*	NA	NA	+	Shamshirgaran et al. (2020)
	*Solanum stramoniifolium*	Ma uek มะอึก	NA	+	+	+	Svobodova et al. (2017)
	*Solanum torvum*	Makhuea puang มะเขือพวง	*+*	+	+	+	Obiang et al. (2019)
Zingiberaceae	*Boesenbergia rotunda*	Krachai กระชาย	*+*	*+*	NA	NA	Atun, Handayani & Rakhmawati (2018)
	*Curcuma longa*	Kamin ขมิ้น	*+*	+	+	NA	Praditya et al. (2019)
	*Zingiber cassumunar*	Plai ไพล	*+*	+	+	NA	Taechowisan, Suttichokthanakorn & Phutdhawong (2018)
	*Zingiber officinale*	Khing ขิง	*+*	+	+	+	Beristain-Bauza et al. (2019)

**Note:**

+, inhibited; −, not inhibited; NA, not analyzed.

When considering selected genera within a family screened for antibacterial activity, particularly prominent species include *Centella asiatica* of Apiaceae (Soyingbe, Mongalo & Makhafola, 2018), *Coccinia grandis* (Laboni et al., 2017), *Momordica charantia* (Villarreal-La Torre et al., 2020) of Cucurbitaceae, *Sesbania grandiflora* of Fabaceae (Anantaworasakul et al., 2017; Noviany et al., 2020), *Cissampelos pareira* of Menispermaceae (Uthpala & Raveesha, 2019), *Solanum* spp. of Solanaceae (Svobodova et al., 2017; Obiang et al., 2019), and *Curcuma longa* (Praditya et al., 2019) and *Zingiber* spp. of Zingiberaceae. Recent publication has rarely investigated all four targeted pathogenic bacteria in a single experimental design. Thus, the evidence is decisive only for the tested antibacterial actions of each plant species. However, some of the selected plant species have demonstrated broad antibacterial activity. For example, among eight bacteria strains tested, fresh *Allium sativum* (Amaryllidaceae) extract had the lowest MIC value of 6.25% (mg/ml) against *S. aureus* (MSSA and MRSA), *E. coli* (ATCC 25922 and MBL), and *K. pneumoniae* (ESBL) (Magryś, Olender & Tchórzewska, 2021). As another example, fruit extract of *Solanum torvum* (Solanaceae) showed potent antibacterial activity against multiple clinical bacterial isolates, with MIC values ranging from 1.25–5 μg/mL (Obiang et al., 2019).

In many cases, the antibacterial activity of a plant extract may be ascribed to predominant essential oils, such as in an essential oil extract of *Ocimum gratissimum* (Lamiaceae) that exhibited MIC values of 1,000 μg/mL against *S. aureus* and *E. coli* (Melo et al., 2019). Fruit peel oil and leaf oil of *Citrus hystrix* (Rutaceae) formulated as oral sprays have demonstrated antibacterial activity against respiratory pathogens, including *S. aureus* ATCC 29213 (Srifuengfung et al., 2020). Other classes of compounds also function as bioactive antibacterial agents, as demonstrated for the phenylbutanoid (*E*)-3-(3,4-dimethoxyphenyl)-4-[(*E*)-3,4-dimethoxystyryl] cyclohex-1-ene isolated from *Z. cassumunar* (Zingiberaceae), which showed high antibacterial activity against *S. aureus* and *E. coli* with MIC values of 16 μg/mL (Taechowisan, Suttichokthanakorn & Phutdhawong, 2018), while also having only weak cytotoxic activity. These findings support that many plant ingredients in Thai food can be taken as supplements for restoring health and can serve as powerful resources for developing antibiotic agents to treat serious and common infectious diseases.

### Antiviral activities

Given the pandemic situation for the past few years, new and effective antiviral agents are needed for the development of vaccines and drugs. Currently available synthetic drugs may have adverse effects or cause drug resistance to nucleoside analogs *via* mutation (Mohan et al., 2020b). For these reasons, plant sources and phytomedicine have gained much interest in relation to antiviral drug discovery. Edible and medicinal plants are a powerful source of bioactive compounds and advantageous in terms of safety. Among the 69 plant species covered here, a few have been well-studied with regard to constituent phytochemicals and have demonstrated broad antiviral activities; these include *Allium sativum* (Amaryllidaceae) (Rouf et al., 2020) and *Curcuma longa* (Zingiberaceae) (Praditya et al., 2019; Jennings & Parks, 2020). Antioxidant and antimicrobial effects have also been reported for species including *Syzygium aromaticum*, *Eryngium* spp., *Cinnamomum* spp., *Curcuma longa*, and *Z. officinale* (Parham et al., 2020). In the current review, we give an update on 17 plant species (12 families) recently published for their antiviral activity against some human viruses (Table 5). In particular, several studies have demonstrated effectiveness of plant extracts and, in some cases, isolated compounds against important enveloped DNA and RNA viruses that cause human diseases such as influenza A, herpesvirus, Dengue virus, Zika virus, and Chikungunya virus, and against the non-enveloped RNA poliovirus. These viruses cause infectious diseases on scales ranging from individual infections and small local outbreaks to pandemics. As regards plant species, *Moringa oleifera* (Moringaceae) (Nasr-Eldin, Abdelhamid & Baraka, 2017; Ashraf et al., 2017; Adamu et al., 2020) and *Piper nigrum* (Piperaceae) (Priya & Kumari, 2017; Nag & Chowdhury, 2020) are of particular interest as they have shown a vaster range of antiviral activities. In the case of *Piper nigrum*, this might be an effect of the bioactive alkaloid piperine or derivatives, which are known for antiviral effects against HSV and the flu virus (Mohan et al., 2020b), while the activity of *Moringa oleifera* may be attributable to isothiocyanate-1, which possess anti-inflammatory properties, or to other constituents (Jaja-Chimedza et al., 2017). Moreover, comparing plant species in Table 5 with Table 1 reveals 14 antiviral plants that have exhibited anti-inflammatory properties, suggesting a synergism between immunomodulatory effects and the inhibition of viral invasion or replication.

**Table 5:** Plants that show antiviral activities.

Family	Botanical name	Local name	Activity	Dose (μg/mL)	References
Apiaceae	*Centella asiatica*	Bua bok บัวบก	Water extract showed anti-herpes simplex virus (HSV1) Water extract showed anti-herpes simplex virus (HSV2)	362.40 298.84	Garber, Barnard & Pickrell (2021)
	*Coriandrum sativum*	Phak chi ผักชี	Aqueous extracts reduced the formation of HSV1 plaques	350	Fayyad, Ibrahim & Yaakob (2017)
Caricaceae	*Carica papaya*	Malako มะละกอ	Fruit pulp extract showed inhibition against the Zika virus Lactic fermented pulp showed inhibition against the Zika virus Leaves extract treated adult dengue patients increased platelet counts compared to placebo group	0.3 4 >1000	Haddad et al. (2020), Sathyapalan et al. (2020)
Cucurbitaceae	*Momordica charantia*	Mara มะระ	Ethanolic extract inhibited human herpes virus-3 (Varicella Zoster virus or HHV-3) Inhibition against the Zika virus	125 507.2	Angamuthu et al. (2019), Vista et al. (2020)
Fabaceae	*Acacia pennata*	Cha om ชะอม	Inhibition against aquatic stages of the dengue virus vector: the 3rd instar larvae and pupae.	>1000	Thongwat, Ganranoo & Chokchaisiri (2017)
	*Leucaena leucocephala*	Kra tin กระถิน	Inhibition against yellow fever virus (BeH111 strain) Inhibition against dengue 1 virus (Hawaii strain)	>1000 >1000	Kaushik et al. (2018)
	*Tamarindus indica*	Ma kham มะขาม	Inhibition against Newcastle disease virus replication	>1000	Okoh et al. (2017)
Lamiaceae	*Ocimum sanctum*	Kaphrao กระเพรา	Crude extract showed highly significant in decreasing the H9N2 virus replication using *in ovo* model.	>1000	Ghoke et al. (2018)
Menispermaceae	*Tiliacora triandra* Diels	Yanang ย่านาง	Ethanolic extract strongly inhibited porcine reproductive and respiratory syndrome virus infectivity in MARC-145 cells	>1000	Arjin et al. (2020)
Moringaceae	*Moringa oleifera*	Ma rum มะรุม	Inhibitory activity against HSV1 and HSV2 Inhibitory activity against Influenza virus Inhibitory activity against Poliovirus	200 0.78–100 >1000	Nasr-Eldin, Abdelhamid & Baraka (2017), Ashraf et al. (2017), Adamu et al. (2020)
Myrtaceae	*Syzygium aromaticum*	Kanplu กานพลู	Inhibition against Newcastle disease virus replication	–	Mehmood, Farooq & Youusaf (2020)
Piperaceae	*Piper nigrum*	Phrik Thai พริกไทย	Inhibitory activity against Vesicular stomatitis Indiana virus Inhibitory activity against Human parainfluenza virus Inhibitory activity of Methyltransferase (PDB id 1L9K) of Dengue and VP35 Interferon Inhibitory Domain (PDB id 3FKE) of Ebola virus	200 600 –	Priya & Kumari (2017), Nag & Chowdhury (2020)
Poaceae	*Cymbopogon citratus*	Ta khrai ตะไคร้	Inhibitory activity against dengue virus serotype 2 (DENV-2)	20	Rosmalena et al. (2019)
Solanaceae	*Capsicum annuum*	Phrik chifah พริกชี้ฟ้า	Inhibitory activity against HSV1 and HSV2	25	Hafiz et al. (2017)
Zingiberaceae	*Boesenbergia rotunda*	Krachai กระชาย	Extract suppressed coronavirus SARS-CoV-2 infectivity Isolated compound cyclohexenyl chalcone derivative Panduatin A suppressed SARS-CoV-2 infectivity	3.62 0.81	Kanjanasirirat et al. (2020)
	*Curcuma longa*	Kamin ขมิ้น	Inhibitory activity against dengue virus serotype 2 (DENV-2)	147	Ichsyani et al. (2017)
	*Zingiber officinale*	Khing ขิง	Gingerenone A inhibited Janus Kinase 2 activity against influenza A virus Inhibitory activity against Chikungunya virus	– 62.5	Wang et al. (2020), Kaushik et al. (2020)

Remarkably, some plant species stand out based on their effectiveness at very low concentrations; these are *Carica papaya* (Caricaceae) (Haddad et al., 2020), *Cissampelos pareira* (Menispermaceae) (Ashraf et al., 2017), and *B. rotunda* (Zingiberaceae) (Kanjanasirirat et al., 2020), and the findings suggest sufficient specificity (extracts were used at concentrations of less than 10 µg/mL) that these might be good candidates for developing antiviral agents and merit further evaluation against a broader group of viruses. Particularly, *B. rotunda* extract and its isolated component panduratin A have promising antiviral activity against SARS-CoV-2 (COVID-19) (Kanjanasirirat et al., 2020). The authors found that infected Vero E6 cells were rapidly suppressed after treatment with extract or panduratin A, which had IC_50_ values of 3.62 μg/mL (CC_50_ = 28.06 μg/mL) and 0.81 μM (CC_50_ = 14.71 μM), respectively. At the pre-entry phase, panduratin A inhibited SARS-CoV-2 infection with IC_50_ of 5.30 μM (CC_50_ = 43.47 μM). However, we have only summarized recent investigations of antiviral activity. Many more plant species on the list might also have potential as sources of antiviral agents, just waiting for other researchers to discover them.

## Conclusions

Plants used as ingredients in Thai food are typically also medicinal plants with applications proven long ago in folk medicine. This review describes the great extent of available information on edible and medicinal plants and isolated molecules from Thai food sources, which until now has existed as scattered pieces of information that have never been combined. The plant list includes diverse families with therapeutic importance supported by the various pharmacological activities, significant bioactive metabolites, and updated anti-inflammatory, antibacterial, and antiviral properties for which evidence has been collected in recent years. Based on the literature, plants used as ingredients in Thai food would be justified as elements of a healthy and functional diet and as sources of up-and-coming drug candidates with lesser toxicity. Many plant species have dual activity, demonstrating both anti-inflammatory and antimicrobial effects towards antibiotic-resistant bacteria and clinically significant viruses. In addition, more than one plant species is generally used for a single dish, as in traditional medicine practice. Hence, the effect of plant-plant combinations should be evaluated so as to enhance health restoration, therapeutic effects, and the development of supplementation and pharmaceutical-related products.

In Thailand, *Andrographis paniculata* (Burm. f.) Wall. ex Nees or Fah Talai Jone, a plant that has been used to treat and relieve common cold symptoms for years, has been included in The National List of Essential Drugs since 1999 and has become of renewed interest because of the COVID-19 pandemic around the globe. Encapsulated powder or extract of *Andrographis paniculata* with its major component, andrographolide, taken as a dose of 180 mg per day for five consecutive days, was recommended by the department of medicinal services for patients with mild symptoms (Mahajaroensiri et al., 2021). Concerning toxicity, a recent research article indicated that *Andrographis paniculata* extract and andrographolide respectively had no toxicity and a favorable toxicity profile in six representative human cell lines from the liver, kidney, intestine, lung, and brain. Both extract and andrographolide possess anti-SARS-CoV-2 activity and should be further investigated for their bioavailability and development for clinical applications as a monotherapy or in combination with other drugs (Sa-Ngiamsuntorn et al., 2021). In addition to that exemplar, this review emphasizes a promising plant list that will be of help in encouraging further investigation into mechanisms, synergy with antibiotics, formulations, physicochemical properties, bioavailability, and clinical research for the practical utilization of bioactive plant products. Moreover, the current review shall aid in the better selection of plant parts and species and promote their evaluability as food ingredients, functional foods, beverages, dietary supplements, and herbal medicines to preserve and increase vitality, slow aging, and promote well-being.

## Additional Information and Declarations

### Data Availability

The following information was supplied regarding data availability: This literature review analyzed previous articles.
